# Human cerebrospinal fluid affects chemoradiotherapy sensitivities in tumor cells from patients with glioblastoma

**DOI:** 10.1126/sciadv.adf1332

**Published:** 2023-10-25

**Authors:** Brett W. Stringer, Manam Inushi De Silva, Zarina Greenberg, Alejandra Noreña Puerta, Robert Adams, Bridget Milky, Michael Zabolocki, Mark van den Hurk, Lisa M. Ebert, Christine Fairly Bishop, Simon J. Conn, Ganessan Kichenadasse, Michael Z. Michael, Rebecca J. Ormsby, Santosh Poonoose, Cedric Bardy

**Affiliations:** ^1^ South Australian Health and Medical Research Institute (SAHMRI), Laboratory for Human Neurophysiology and Genetics, Adelaide, SA, Australia.; ^2^ Flinders Health and Medical Research Institute, College of Medicine and Public Health, Flinders University, Adelaide, SA, Australia.; ^3^ Centre for Cancer Biology, University of South Australia and SA Pathology, Adelaide, SA, Australia.; ^4^ Cancer Clinical Trials Unit, Royal Adelaide Hospital, Adelaide, SA, Australia.; ^5^ Adelaide Medical School, University of Adelaide, Adelaide, SA, Australia.; ^6^ Flinders Medical Centre, SA Health, Adelaide, SA, Australia.

## Abstract

Cancers in the central nervous system resist therapies effective in other cancers, possibly due to the unique biochemistry of the human brain microenvironment composed of cerebrospinal fluid (CSF). However, the impact of CSF on cancer cells and therapeutic efficacy is unknown. Here, we examined the effect of human CSF on glioblastoma (GBM) tumors from 25 patients. We found that CSF induces tumor cell plasticity and resistance to standard GBM treatments (temozolomide and irradiation). We identified nuclear protein 1 (NUPR1), a transcription factor hampering ferroptosis, as a mediator of therapeutic resistance in CSF. NUPR1 inhibition with a repurposed antipsychotic, trifluoperazine, enhanced the killing of GBM cells resistant to chemoradiation in CSF. The same chemo-effective doses of trifluoperazine were safe for human neurons and astrocytes derived from pluripotent stem cells. These findings reveal that chemoradiation efficacy decreases in human CSF and suggest that combining trifluoperazine with standard care may improve the survival of patients with GBM.

## Teaser

The human brain biochemistry causes cancer cell plasticity and treatment resistance but also provides therapeutic opportunities.

## INTRODUCTION

Brain cancers kill more children and adults under 40 years of age than any other cancer (*1*). The most common primary malignancy of the central nervous system (CNS)—glioblastoma (GBM)—has no cure. The current standard of care for GBM is maximal surgical resection, followed by fractionated radiotherapy (25 to 30 × 2 Gy) with concurrent and subsequent oral temozolomide (TMZ) chemotherapy for 6 months (*2*, *3*). However, despite this aggressive treatment, GBM tumors almost invariably recur and prove fatal (*4*), indicating that treatment-resistant malignant cells remain. GBM median survival is <15 months (*5*), and alternative therapies such as immunotherapies have not improved this outcome (*6*, *7*). Therefore, identifying therapies that can contribute to extending the survival rate of GBM is a major unmet need (*8*), and more accurate preclinical models are required to expedite this translational goal.

Cancer cells in the CNS are often clinically resistant to therapies that eradicate cancers elsewhere in the body (*9*, *10*). Previous scrutiny of the effect of blood, lymphatic vasculature, angiogenesis factors, cytokines, immune cells, and extracellular matrix on cancer cells in the GBM tumor niche (*11*) demonstrates the important role that the microenvironment plays in cancer progression, plasticity, and treatment resistance (*12*). However, the primary base of the CNS microenvironment is cerebrospinal fluid (CSF), and its effect on cancer progression and treatment resistance is poorly understood. Physically, CSF provides buoyancy for the brain, and, biochemically, it provides brain cells with nourishment and maintains a stable interstitial neuronal milieu (*13*, *14*). CSF is produced by the choroid plexus ependymal cells in the ventricles, which selectively filter nutrients and electrolytes from blood plasma. In addition, CSF is rich in trophic factors synthesized in the choroid plexus, including brain-derived neurotrophic factor (BDNF), glia-derived neurotrophic factor (GDNF), nerve growth factor, fibroblast growth factor (FGF), epithelial growth factor (EGF), transforming growth factor–β, platelet-derived growth factor, insulin growth factor (IGF), vascular endothelial growth factor, and the homeoprotein Otx2 (*14*–*16*). CSF biochemistry is thus different from plasma (*14*) and creates a unique extracellular environment in the CNS that is not found anywhere else in the body. CSF composition also differs from the defined serum-free tissue culture medium used as the gold standard in GBM preclinical studies. This glioma medium (GM) was designed to support the growth of tumor initiating GBM stem cells (*17*–*19*) and mimics the microenvironment within a large tumor mass rather than the CSF-infused human brain microenvironment.

Tumors in contact with the lateral ventricle can disrupt the ependymal wall and increase the influx of CSF toward the tumor (*20*). Similarly, following neurosurgery, the void left in place of the tumor mass naturally fills with CSF, and the GBM cells that cannot be surgically removed at the margins of the resection cavity are flushed with CSF (*21*). Patients with GBM tumors located in the vicinity of the CSF-filled ventricles have a lower survival rate (*22*, *23*), suggesting that exposure to CSF promotes cancer cell malignancy. However, the mechanisms underlying CSF-induced cellular plasticity and whether it influences treatment efficacy are unknown.

We tested the hypothesis that CSF exposure changes the molecular profile of GBM tumor cells and influences treatment sensitivities. We thoroughly examined the effects of fresh human CSF on GBM tumor cells derived from 25 patients. We found that brief (3 to 7 days) exposure to human CSF can shift the molecular identity of single cells, protecting them from standard chemoradiotherapies. We then identified several molecular pathways that underlie CSF-induced plasticity. Interfering with one of these pathways, *NUPR1*-regulated ferroptosis, with a repurposed drug [trifluoperazine (TFP)] improved the efficacy of current treatments on CSF-exposed GBM cells. We observed that the same doses effective in vitro had no lasting side effects on the survival and electrophysiology of human cortical and midbrain neurons and astrocytes (ACs). Our findings highlight the unique and previously unknown influence of CSF on GBM cells with a large cohort of GBM patient tumors and provide preclinical evidence that TFP may improve the treatment of GBM.

## RESULTS

To investigate the effect of human CSF on the phenotype of GBM cells, we established a collection of early passage cell lines from 25 GBM patient tumor tissue biopsies resected as part of the standard neurosurgical treatment. All were from isocitrate dehydrogenase (IDH)–wild-type GBM, 23 were from treatment naïve tumors, and 2 were from recurrent GBMs initially treated by surgical resection and adjuvant radiotherapy and chemotherapy. The assembled cell lines exhibited a variety of morphologies (size, shape, and heterogeneity), proliferation rates, genetic and transcriptomic profiles, and sensitivity to TMZ and irradiation (table S1 and data presented herein). This diversity effectively models the substantial intra- and interpatient diversity of GBM tumors.

GBM is a notoriously plastic cancer, a trait underlying its therapeutic resistance (*24*). However, the influence of CSF on GBM plasticity and treatment sensitivity is unknown. This oversight may be due, at least in part, to the relatively small volume of CSF in the human CNS (total of 125 ml in adults, recycled four times per day) and the challenging logistics of obtaining sufficient CSF for rigorous preclinical investigations. To mitigate this challenge, we used fresh CSF collected from otherwise healthy individuals with normal pressure hydrocephalus or idiopathic intracranial hypertension as part of the management of these conditions (*25*, *26*). Although much more challenging to obtain in sufficient volume, we confirmed in a subset of experiments that CSF collected from patients with GBM had similar effects on cancer cells than normal CSF. Glucose concentration, protein concentration, and osmolality were measured for each sample to identify potential outliers and control the samples’ quality. The CSF samples from several patients were pooled to avoid patient-specific bias (table S2). Patient-derived GBM cells were cultured in either 100% human CSF or defined GM for comparison. GM consisted of Dulbecco’s modified Eagle medium (DMEM)/F12 supplemented with N2 Supplement-A (N2A), SM1 containing vitamin A, EGF (5 ng/ml), and FGF-basic (5 ng/ml). Serum-free GM was designed to better preserve the phenotype and genotype of primary tumors than serum-containing medium (*17*) and was used in almost all preclinical research studies that culture human GBM cells from patient biopsies (*18*).

### GBM cells are larger and elongated in CSF

**Fig. 1.**
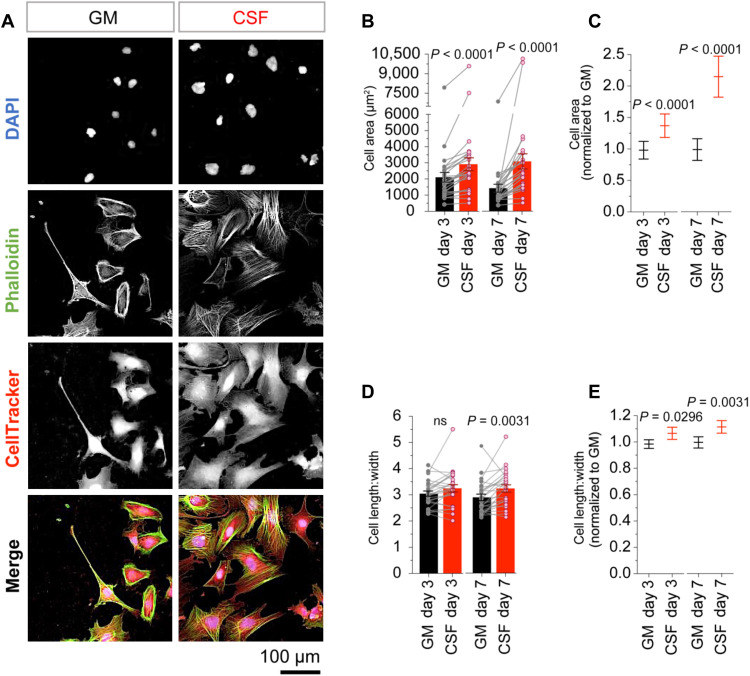
GBM cells are larger and elongated in CSF. (**A** to **E**) Morphology analysis of patient-derived GBM cells cultured in CSF or standard GM for 3 or 7 days. (A) Representative images of GBM cells (SB2b) cultured in GM and after 3 days’ exposure to CSF. Cells are stained with DAPI, phalloidin iFluor 488, and CellTracker Deep Red. (B) Cell area and (D) cell length:width determined using Harmony software analysis of CellTracker Deep Red–stained cells (*n* > 5000 per cell line). Individual data points represent the mean of six replicates for each of 25 GBM cell lines. Bar graphs represent the means ± SEM of all 25 GBM cell lines. Significance was determined using paired, two-way Wilcoxon tests. (C) Cell area and (E) cell length:width normalized to GM. Graphs represent the means ± SEM of all 25 GBM cell lines. Significance was determined using paired, two-way Wilcoxon tests. ns, not significant.

To determine whether exposure to human CSF had any effect on GBM cells, we first looked at possible induced changes in cell morphology. Cells were cultured on growth factor–reduced Matrigel, with six replicates of each cell line under both conditions. Cells were cultured for 3 or 7 days in CSF or GM and stained with 4′,6-diamidino-2-phenylindole (DAPI), CellTracker Deep Red and phalloidin iFluor 488 to visualize and quantitate cell and nucleus length, width, and area, using an Operetta CLS high-content confocal imaging system. Cells from all 25 GBM cell lines were larger in CSF compared to GM (Fig. 1, A to C, and fig. S1, A, B, and M). This difference was apparent after 3 days of culture and increased further for some cell lines following an additional 4 days’ exposure to CSF (Fig. 1, B and C, and fig. S1, A, B, and M). We observed an increase in both cell length and cell width (fig. S1, C to J and M). However, the increase in cell length was greater than the increase in cell width; thus, GBM cells were more elongated in CSF than in GM (Fig. 1, A, D, and E, and fig. S1, K to M). We saw similar changes in nuclear morphology, with GBM cell nuclei appearing larger and more elongated in CSF (Fig. 1A and fig. S1M). The cell cytoplasm compartment (excluding the nucleus) also appeared larger and more elongated. We also noticed that microtubules of some cell lines stained more prominently in CSF (Fig. 1A and fig. S1M). Similar changes in morphology were observed when GBM cells were exposed to CSF collected from a patient with GBM (fig. S2, A to H). In summary, we observed uniform effects of CSF on GBM cell morphology, which became larger and more elongated after only 3 days’ exposure to CSF.

### GBM cells are more resistant to TMZ and irradiation in CSF

**Fig. 2.**
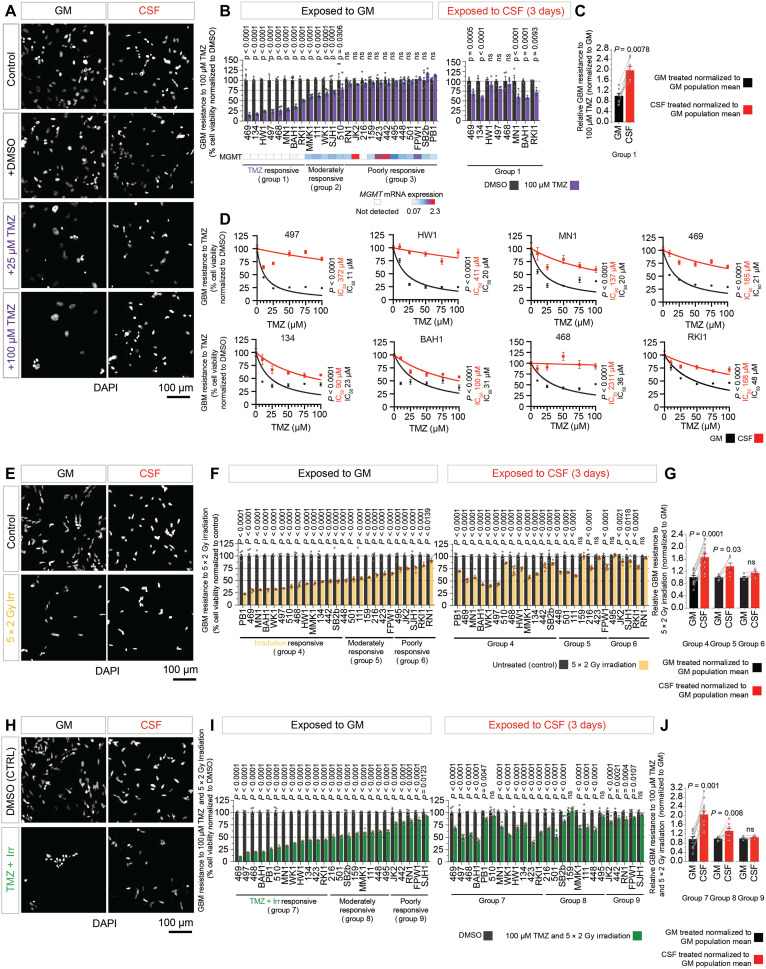
Patient-derived GBM cell lines display heterogeneous responses to standard therapeutics. (**A** to **J**) Cytotoxicity analysis of patient-derived GBM cells cultured in CSF or GM for 3 days before treatment. Representative images of (A) cell lines untreated or treated with 1:2000 dimethyl sulfoxide (DMSO) control and 25 and 100 μM TMZ for 7 days (SANTB00497); (E) untreated or treated with five fractions of 2-Gy irradiation over 5 days (SANTB00469); (H) treated with 1:2000 DMSO or five fractions of 2-Gy irradiation and 100 μM TMZ for 5 and 7 days, respectively (SANTB00469). Cells are stained with DAPI. Responsiveness of 25 patient-derived GBM cell lines exposed to (B) 1:2000 DMSO or 100 μM TMZ for 7 days, (F) five fractions of 2-Gy irradiation for 5 days, and (I) 1:2000 DMSO or five fractions of 2-Gy irradiation and 100 μM TMZ. Individual data points represent six replicates for each GBM cell line normalized to the control. Bar graphs represent the means ± SEM. Significance determined using two-way analysis of variance (ANOVA). Fold change of (C) TMZ-responsive lines (group 1, <50% cell survival); (G) lines responsive (group 4), moderately responsive (group 5, 50 to 75% cell survival), or unresponsive (group 6, >75% cell survival) to irradiation; and (J) lines responsive (group 7), moderately responsive (group 8), or unresponsive (group 9) to combination treatment. Cell viability in GM and CSF normalized to mean population survival in GM. Individual points represent the mean of six replicates for each GBM cell line. Significance determined using two-way paired, nonparametric Wilcoxon test. (D) Dose response of TMZ responsive lines (group 1) treated with 1:2000 DMSO (0 μM) and 12.5, 25, 50, and 100 μM TMZ. Cell counts normalized to 0 μM GM or CSF. DAPI-stained cells analyzed using Harmony. Significance and IC_50_ determined by nonlinear regression. ns defined as *P* > 0.05.

TMZ and irradiation are the current standard of care following surgical tumor resection (*2*, *27*). Therefore, we investigated the effect of CSF exposure on the efficacy of TMZ and irradiation (separately and in combination) to kill patient-derived tumor cells in vitro. We first screened the 25 GBM cell lines for their response to TMZ in GM. We observed a cell line–specific response range to 7 days’ TMZ treatment (100 μM), consistent with the heterogeneity of clinical response (Fig. 2, A and B, and fig. S3, A and B). Each patient cell line was stratified into one of three groups based on cell viability after 7 days of TMZ treatment in GM. The GBM cell lines in group 1 (*n* = 8) were the most TMZ-responsive, with more than half the cells killed by the treatment (Fig. 2, A and B). O^6^-methylguanine-DNA methyltransferase (MGMT) expression (arising from demethylation of the *MGMT* promoter) previously has been correlated with resistance to TMZ (*28*). Consistent with these findings, none of the highly TMZ-responsive tumor lines in group 1 expressed *MGMT* mRNA (Fig. 2B). In group 2, the GBM cell lines (*n* = 5) were moderately responsive to TMZ with 50 to 90% viability (Fig. 2B) and expressed moderate levels of *MGMT*. The remaining 12 lines in group 3 were poorly responsive with >90% viable cells despite the same treatment (Fig. 2B) and expressed *MGMT* at variable levels, but overall higher than in the other two groups.

We then compared the dose-dependent effect of TMZ (0, 10, 25, 50, 75, and 100 μM) on the eight most TMZ-responsive GBM cell lines (group 1) after 3 days’ exposure to CSF. The TMZ resistance of all eight cell lines increased significantly in CSF, compared to their response in GM (Fig. 2, C and D, group 1; and fig. S3, A and B), with a 3- to 64-fold increase in the median inhibitory concentration (IC_50_) of TMZ (Fig. 2D).

We then investigated the response of GBM cell lines to irradiation (Fig. 2, E to G, and fig. S3, A to D and F). Using the same criteria as for TMZ, we found that 14 GBM cell lines were responsive to irradiation in GM (Fig. 2, E and F, group 4), 6 GBM lines were moderately responsive (Fig. 2F, group 5), and 5 GBM lines were poorly responsive (Fig. 2F, group 6). GBM cell lines responsive (Fig. 2G, group 4) and moderately responsive (Fig. 2G, group 5) to irradiation in GM became significantly more resistant following 3 days’ exposure to CSF (60% versus 30% increase, respectively).

We then compared the response of GBM cells to combined chemoradiotherapy, treating cells with TMZ (100 μM) for 7 days and 5 × 2 Gy fractions of radiation over the final 5 days of TMZ treatment (Fig. 2, H to J, and fig. S3E). When cultured in GM, the cells again showed a range of resistance to treatment (Fig. 2, H and I, and fig. S3A); 12 cell lines were responsive to TMZ and irradiation (Fig. 2, H and I, group 7); 8 cell lines were moderately responsive (Fig. 2I, group 8); and 5 cell lines were poorly responsive (Fig. 2I, group 9). The response of each cell line to the combination of TMZ and irradiation was not additive, indicating that cell killing by the two therapies did not occur through exclusive pathways (fig. S3A).

When the cells were exposed to CSF for 3 days before treatment and maintained in CSF during the 7-day combined treatment, GBM cell lines in groups 7 and 8 were significantly more resistant to chemoradiotherapy (TMZ and irradiation). The effect was more pronounced in group 7 than group 8 (100% versus 35% increase, respectively; Fig. 2J).

In summary, following exposure to CSF, GBM tumor lines from a diverse panel of patients become largely resistant to TMZ, irradiation, and combined chemoradiotherapy, despite the relatively high efficacy of the same treatments under classical GM conditions. We then sought to understand what mechanisms underlie CSF-induced changes in therapeutic sensitivity.

### GBM cells become more quiescent and less proliferative in CSF

**Fig. 3.**
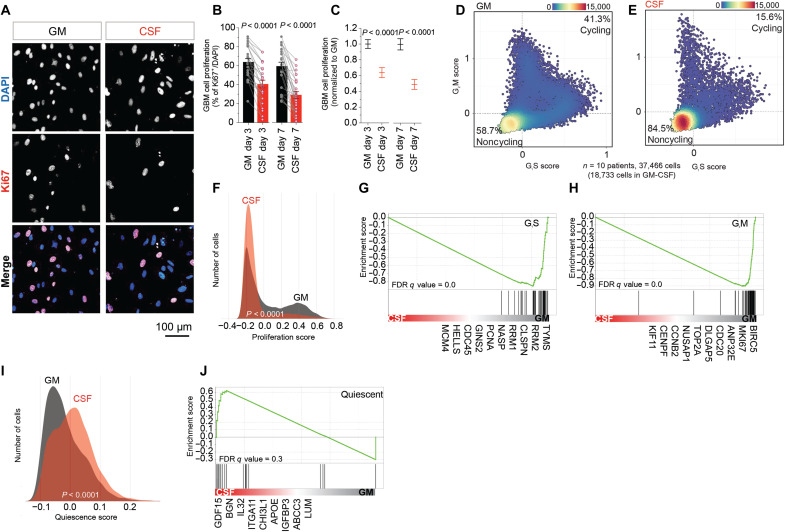
GBM cells become less proliferative and more quiescent in CSF. (**A** to **J**) Proliferation analysis of patient-derived GBM cells cultured in CSF or GM for 3 or 7 days. (A) Representative images of GBM cells (SANTB00442) cultured in GM and after 3 days’ exposure to CSF. Cells are stained with DAPI and for Ki67. (B) Percentage of proliferating cells determined using Harmony software analysis of Ki67^+^ and DAPI^+^ cells. Individual data points represent six replicates for each GBM cell line. Bar graphs represent the means ± SEM of all 25 GBM cell lines. Significance determined using paired, two-way Wilcoxon tests. (C) Percentage of proliferating cells in GM and CSF normalized to GM. (D) to (J) Single-cell RNA sequencing (scRNA-seq) analysis of 10 patient-derived GBM cell lines (*n* = 18,733 cells in GM-CSF) cultured in GM or CSF for 3 days. (D) and (E) Scatter plots representing the percentage of cycling (G_1_S or G_2_M > 0) and noncycling cells (G_1_S and G_2_M < 0) in GM and CSF. G_1_S and G_2_M scores were calculated for each individual cell in the dataset using gene lists by Tirosh *et al.* (*31*). Density plots showing (F) proliferation and (I) quiescence scores of cells in GM and CSF. The proliferation score represents an average of G_1_S and G_2_M scores. Quiescence score was calculated using gene lists by Atkins *et al.* (*30*). Significance was calculated using a two-way, unpaired Mann-Whitney test. Gene set enrichment analyses (GSEAs) of (G) and (H) G_1_S and G_2_M genes and (J) quiescence genes. Genes differentially expressed in CSF and GM were scored as sign(log_2_FC)* − log_10_(adjusted *P*). GSEA was performed using preranked GSEA with default settings (permutations; 1000, enrichment statistic; weighted). ns defined as *P* > 0.05. FDR, false discovery rate.

Most chemoradiotherapies are more effective at killing actively dividing cells (*29*). Therefore, we first asked whether the greater viability of GBM cells in CSF after TMZ and irradiation treatments was concurrent with reduced cell proliferation. We compared cell numbers after 3 and 7 days’ growth in CSF or GM and the percentage of cycling cells as indicated by Ki67 immunofluorescence. Most GBM cell lines proliferated slower in CSF (Fig. 3, A and B, and fig. S4, C to E). The percentage of Ki67^+^ cycling cells was significantly lower after 3 and 7 days’ growth in CSF for most GBM cell lines (Fig. 3, B and C). However, it is worth noting that a few lines grew just as well in CSF (e.g., RKI1 and 448; fig. S4, C and D). GBM cells exposed to CSF collected from a patient with GBM similarly proliferated slower (fig. S2, I and J). Overall, 24 of 25 lines grew less rapidly after 3 days in CSF, and 22 of 25 lines grew slower after 7 days.

When proliferating, cells spend time in sequential phases of the cell cycle, including cell growth (gap 1: G_1_ phase), DNA replication (synthesis: S phase), and growth and preparation for mitosis (gap 2: G_2_ phase), before dividing (mitosis: M phase). During the G_1_ phase, some cells can temporarily or permanently exit the cell cycle to enter a quiescent state (resting: G_0_ phase). The specific phases are not usually morphologically distinguishable. Therefore, to gain further insights into the proportion of cells in specific cycling phases, we performed single-cell RNA sequencing (scRNA-seq; *n* = 37,466 viable single cells) with 10 GBM cell lines, cultured for 3 days in either CSF or GM (fig. S5, A to I). We determined the cycling phase using the mRNA expression of defined gene sets (*30*, *31*). For each cell, we determined a G_1_S score (DNA synthesis and replication for G_1_ and S phases combined) and a G_2_M score (mitosis for G_2_ and M phases combined). Low G_1_S and G_2_M scores indicated a noncycling G_0_ phase. We then used the average of G_1_S and G_2_M scores to determine a proliferation score per cell and the expression of known quiescent genes for a quiescence score. These aggregated metrics clearly showed that GBM cells in CSF spend more time in noncycling (Fig. 3, D and E), nonproliferating (Fig. 3F), and quiescent (Fig. 3I) states, confirmed by enrichment of the corresponding gene sets (Fig. 3, G, H, and J). Similarly, uniform manifold approximation and projection (UMAP) analysis showed a greater percentage of noncycling cells in each cell line in CSF (fig. S4A) as well as a lower percentage of cells expressing *MKI67* in CSF (fig. S4B). In summary, our immunocytochemistry and scRNA-seq analyses showed that, overall, most GBM cells proliferate at a slower rate in CSF and CSF promoted quiescent cell states but was not growth limiting.

### GBM cells shift toward a mesenchymal-like state in CSF

**Fig. 4.**
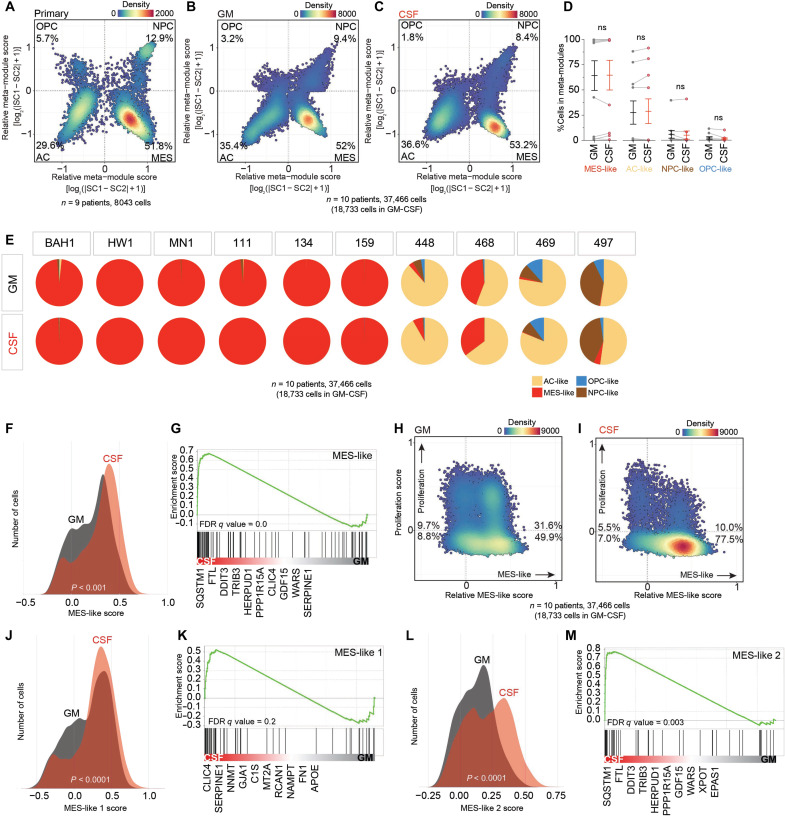
GBM cells shift toward an MES-like state in CSF. (**A** to **H**) scRNA-seq analysis of changes in transcriptomic state using 10 patient-derived GBM cell lines cultured in GM or CSF for 3 days (*n* = 18,733 cells in GM-CSF). Density plots showing (A) MES-like, (E) MES-like 1, and (F) MES-like 2 scores of cells in GM and CSF. MES-like, MES-like 1, and MES-like 2 states were defined using gene lists by Neftel *et al.* (*34*). GSEA of (B) MES-like, (G) MES-like 1, and (H) MES-like 2 genes. Differential expression (CSF versus GM) was performed on a downsampled dataset (*n* = 1400 cells per patient, 700 cells per condition). Relatively up-regulated genes in CSF and GM were scored as sign(log_2_FC)* − log_10_(adjusted *P*). GSEA was performed using preranked GSEA with default settings (permutations; 1000, enrichment statistic; weighted). (C and D) Scatter plots represent the percentage of proliferative, nonproliferative, MES-like, and non–MES-like cells in GM and CSF. Scatter plots represent the percentage of MES-like, AC-like, NPC-like, and OPC-like cells in (**I**) nine patient tumors (*n* = 8043 cells), (**J**) GM, and (**K**) CSF. AC-like, NPC-like, and OPC-like scores were defined using gene lists by Neftel *et al.* (*34*). (**L**) Proportion of MES-like, AC-like, NPC-like, and OPC-like cells in GM and CSF. Individual points represent the 10 patient cell lines with error bars representing the means ± SEM of the population. Significance was determined using two-way, paired Wilcoxon tests. (**M**) Pie charts show the proportion of MES-like, AC-like, NPC-like, and OPC-like cells in GM and CSF for individual cell lines. ns defined as *P* > 0.05.

Tumor cell quiescence and resistance to chemotherapies are often associated with specific cell identities such as mesenchymal (MES) states (*30*, *32*, *33*). GBM cell heterogeneity has been reduced to four main cell types by single-cell transcriptomic analysis (*34*): neural progenitor cell (NPC)–like, oligodendrocyte precursor cell (OPC)–like, AC-like, and MES-like states (Fig. 4A). This single-cell transcriptomic classification largely aligns with the classical classification of bulk tumor types (proneural, classical, and MES) (*35*, *36*) but more accurately reflects intratumor heterogeneity and the dynamic nature of GBM cell identity (*37*, *38*). Therefore, we asked whether short CSF exposure could shift GBM cell identity and molecular states.

We compared the transcriptome (scRNA-seq) of patient-derived GBM cells after 3 days’ exposure to CSF or GM to determine the single-cell molecular identity, as defined by Neftel *et al.* (*34*) (Fig. 4 and fig. S6). In both CSF and GM, our cultured GBM cells (10 patients with GBM; 37,466 viable single cells) were very similar to the single cell–type composition reported by Neftel *et al.* (*34*) for cells from primary GBM tumors (9 patients with GBM; 8043 viable single cells), suggesting that our low-passage patient-derived cell lines represent clinically relevant tumor profiles (Fig. 4, A to C). Despite the predominant overlap in the two independent datasets, we saw a slightly higher percentage of AC-like cells and a slightly lower percentage of OPC- and NPC-like cells in our samples compared to Neftel *et al*. (*34*), which most likely reflects patient heterogeneity (Fig. 4, A to C). Six of our sequenced GBM cell lines, in both CSF and GM, consisted almost entirely of MES-like cells (Fig. 4E and fig. S6G), as previously reported in primary tissue (fig. S6J). In comparison, the other four GBM cell lines mainly consisted of AC-like cells (Fig. 4E and fig. S6G) and various proportions of the other cell types (OPC, NPC, and MES-like cells), which is also seen in freshly resected tumors (fig. S6J).

On average, we observed no significant changes in the composition of individual GBM cell types (AC, MES, OPC, and NPC) when exposed to CSF for 3 days (Fig. 4, D and E, and fig. S6G). However, we saw a statistically significant increase in the MES-like score of GBM cells in response to CSF (Fig. 4, F, H, and I, and fig. S6C) and a corresponding enrichment of MES-like gene expression (Fig. 4G). We also noted a significantly pronounced increase in MES-like 2 scores and gene enrichment in CSF relative to MES-like 1 scores (Fig. 4, J to M). In summary, we found that short exposure to CSF shifted GBM cells toward more MES states.

### *NUPR1* and other potential therapeutic targets are up-regulated upon exposure to CSF

**Fig. 5.**
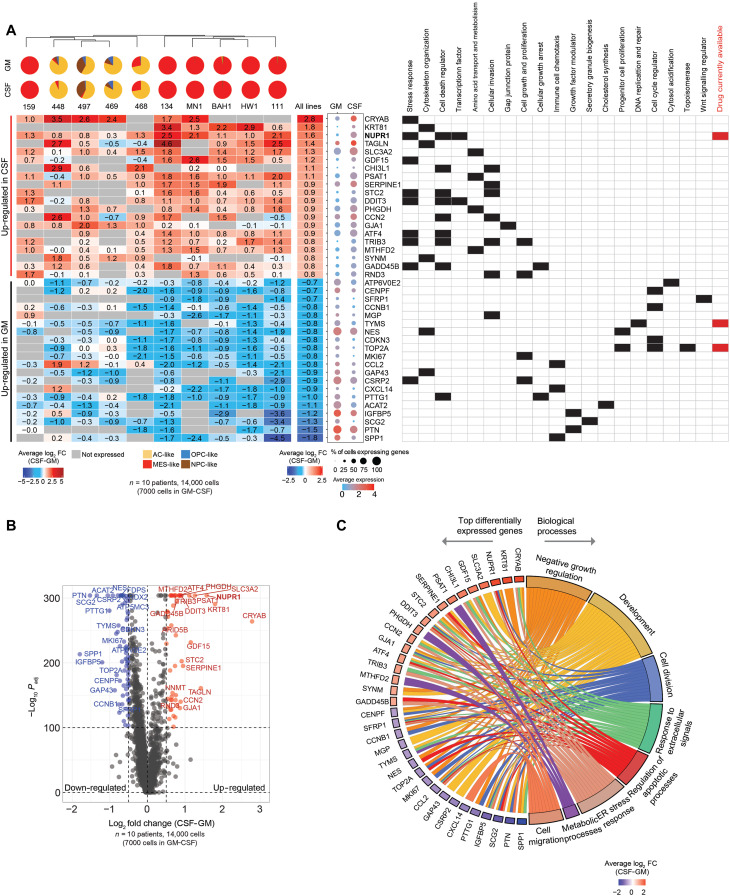
NUPR1 and other potential therapeutic targets are up-regulated upon exposure to CSF. (**A** and **B**) Differential gene expression analysis for GBM cell lines cultured in GM and CSF for 3 days (*n* = 7000 cells in GM-CSF). (A) Heatmap shows the average log_2_ fold change of the top 20 highly expressed genes in CSF and GM. Differential gene expression analysis between CSF and GM was performed for individual cell lines and all cell lines together. Positive (red) average log_2_ fold change represents genes highly expressed in CSF or relatively down-regulated in GM. Negative (blue) average log_2_ fold change represents genes highly expressed in GM or relatively down-regulated in CSF. Dot plot represents the average expression and proportion of cells expressing the top 20 highly expressed genes in GM and CSF. The table represents the functional role of the top genes in GM and CSF. (B) Volcano plot showing genes that are differentially expressed (in red: −log_10_(adjusted *P*) > 100, log_2_(FC) > 0.5; in blue: −log_10_(adjusted *P*) > 100, log_2_(FC) < −0.5) between GBM cells in CSF relative to GM. *P* value adjustment was performed using Bonferroni correction. The top 20 relatively up-regulated (red) and down-regulated (blue) genes are labeled. (**C**) Chord plot shows association of the top differentially expressed genes with GO biological processes. GO overrepresentation analysis was performed using clusterProfiler. Louvain clustering was then applied to significant GO terms (adjusted *P* < 0.05) using the simplifyEnrichment package. New biological process categories were applied for the eight major clusters based on the enriched GO terms. ER, endoplasmic reticulum.

Lower cancer survival is associated with quiescent and MES tumor cell states and underlying resistance to chemotherapies (*30*, *32*, *33*, *39*). We demonstrated an increase in quiescent MES cell states resistant to chemoradiotherapies upon short exposure to human CSF. Therefore, revealing the molecular pathways that drive the response to CSF exposure may indicate potential therapeutic targets. Differential gene expression analysis of our scRNA-seq dataset (Fig. 5, A and B, and fig. S7) showed that many of the top 20 up-regulated and down-regulated genes in CSF have roles in the organization of the cytoskeleton, response to stress, amino acid transport and metabolism, or modulation of growth factor activity, all plausible mediators of the phenotypic changes we observed in GBM cells in response to CSF (Fig. 5, A and C, and fig. S8).

**Fig. 6.**
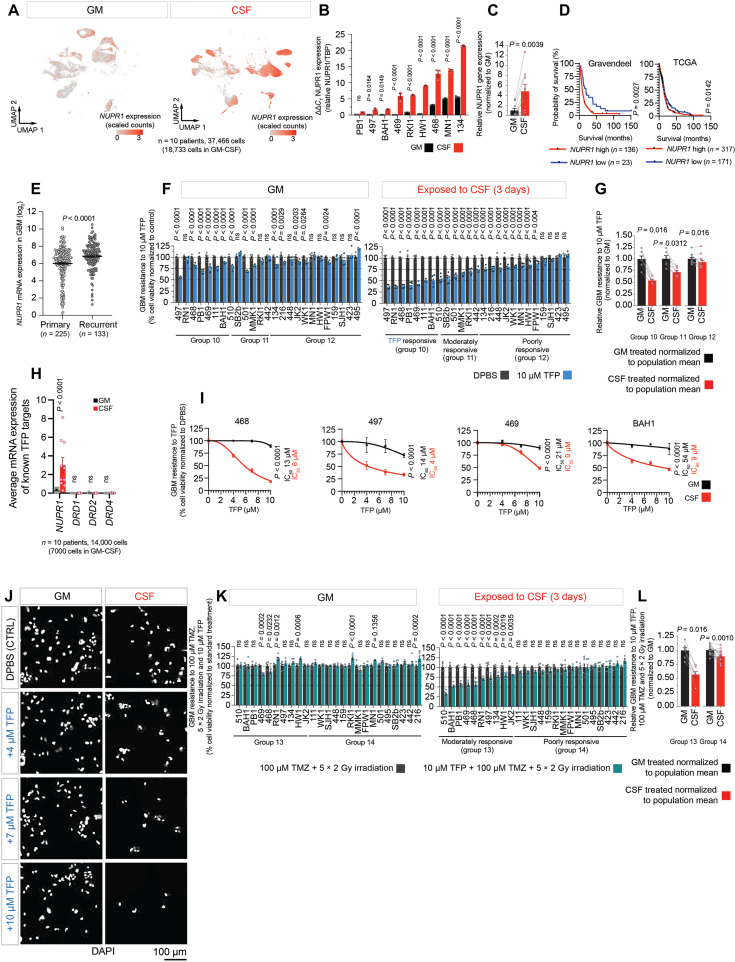
Targeting NUPR1 with repurposed TFP reduces cell survival in CSF. (**A**) UMAP showing *NUPR1* expression in 10 GBM cell lines (*n* = 18,733). (**B**) *NUPR1* expression in cell lines (*n* = 8) cultured in GM or CSF for 3 days measured by reverse transcription quantitative polymerase chain reaction (PCR). Individual data points, replicate measurements. Bar graphs, means ± SEM. Significance determined using two-way ANOVA. (**C**) Fold change in *NUPR1* expression shown in (B). *NUPR1* expression in CSF normalized to GM. (**D**) Kaplan-Meier survival curves showing overall survival versus *NUPR1* expression for GBM. Datasets from GlioVis (*70*). “NUPR1 high” and “NUPR1 low” defined by optimum cutoff. (**E**) *NUPR1* expression in primary and recurrent GBM. CGGA RNA-seq dataset from GlioVis. Scatter plots, means ± SEM. Significance determined using unpaired *t* test with Welch’s correction. Responsiveness of 25 GBM cell lines to (**F**) Dulbecco’s phosphate-buffered saline (DPBS) or TFP for 24 hours or (**K**) irradiation and TMZ or irradiation, TMZ and TFP. Fold change of cell lines (**G**) responsive (group 10), moderately responsive (group 11), or unresponsive (group 12) to TFP and (**L**) moderately responsive (group 13) or unresponsive (group 14) to combination treatment with TFP. Cell viability in GM and CSF normalized to mean population survival in GM. (**H**) Bar graph showing expression of TFP targets from the scRNA-seq dataset. Individual data points, mean expression (*n* = 10). Bar graphs, means ± SEM. Significance determined using two-way ANOVA. (**J**) Representative images of DPBS- and TFP-treated cells (SANTB00468). (**I**) Dose response of TFP-responsive lines (group 10) to TMZ. (F and K) Individual data points represent six replicates of each cell line normalized to control. Bar graphs, means ± SEM. Significance determined using two-way ANOVA. (I) Individual points and error bars, means ± SEM. Significance and IC_50_ determined by nonlinear regression. ns indicates *P* > 0.05.

Gene ontology (GO) analysis showed that the biological processes most significantly affected by these gene expression changes include the cellular response to extracellular signals, cell metabolism, the endoplasmic reticulum stress response, cell division, negative regulation of cell growth, regulation of cell death, development, and migration (Fig. 5C and fig. S8). While some of the most differentially regulated genes are associated with a single biological process, notably the metabolic genes *MTHFD2*, *PHGDH*, and *PSAT1*, and the developmental genes *CSRP2*, *GAP43*, *MGP*, and *SLC3A2*, 10 of them (*CCL2*, *SFRP1*, *DDIT3*, *IGFBP5*, *GJA1*, *NUPR1*, *ATF4*, *CRYAB*, *SCG2*, and *STC2*) are associated with at least four different biological processes (Fig. 5C and fig. S8). Given their broad impact on cell activity, these latter genes may be important nodes of the integrated response of GBM cells to CSF. As a result, they may represent potential therapeutic targets for GBM. However, a search of the literature identified drugs targeting only 3 of the 40 listed genes (*NUPR1*, *TYMS*, and *TOP2A*) (Fig. 5A), suggesting limited therapeutic options. These results provide an exciting resource for guiding future research opportunities. However, here, we decided to focus on one of the three readily druggable targets: *NUPR1*, a gene encoding the transcriptional regulator nuclear protein 1 (NUPR1), the third most up-regulated gene in our analysis (Fig. 5B). We chose *NUPR1* for several reasons: (i) *NUPR1* is a transcription factor, so its increased expression is likely to coordinate a broad network of responses; (ii) *NUPR1* was up-regulated in response to CSF in all 10 GBM cell lines we sequenced (Figs. 5A and 6, B and C); (iii) *NUPR1* was a top 20 up-regulated gene in six cell lines (fig. S7A); (iv) *NUPR1* was expressed by most of the sequenced GBM cells in response to CSF, particularly those with a predominant MES-like cell state (Fig. 6A and fig. S9A); (v) *NUPR1* has been implicated in chemotherapy resistance in other cancers (*40*), as an inhibitor of ferroptosis (*41*); (vi) greater *NUPR1* expression in GBM and recurrent GBM tumors is associated with reduced overall clinical survival (Fig. 6, D and E, and fig. S9B); and, most importantly, (vii) NUPR1 is a readily druggable target. A clinically approved agent, trifluoperazine (TFP), can inhibit NUPR1 and is permeable to the blood-brain barrier. Thus, investigating CSF-induced NUPR1 pathways may provide a tractable rapid path to translation.

### A repurposed psychiatric agent, TFP, kills patient-derived GBM cells in human CSF

We hypothesized that CSF-induced NUPR1 pathways play a role in treatment resistance in GBM cells and sought to preclinically investigate the therapeutic benefit of NUPR1 inhibitors. TFP inhibits NUPR1, *K*_d_ = 5.2 μM (*42*, *43*). We treated all 25 of our GBM cell lines with 10 μM TFP in both CSF and GM, anticipating that TFP might reduce GBM cell viability in CSF. This is indeed what we saw (Fig. 6, F and G). We found that 7 of 25 GBM cell lines were highly TFP-responsive in CSF (<50% viable cells compared to the nontreated control; Fig. 6F, group 10), 6 of 25 were moderately responsive (50 to 90% viable cells; Fig. 6F, group 11), and 12 of 25 were poorly responsive (>90% viable cells; Fig. 6F, group 12). TFP targeted specifically GBM cells in CSF. We observed a significantly stronger GBM cell–killing effect of TFP in CSF compared to GM in all three groups, even the group defined as relatively poorly responsive (Fig. 6G). To investigate further the GBM cell sensitivity to TFP in CSF, we performed a dose-response analysis (0, 4, 7, and 10 μM TFP) using four TFP-sensitive GBM cell lines (Fig. 6, I and J, and fig. S10G). Our analysis showed the IC_50_ of TFP in CSF ranged between 4 and 9 μM, on average, >3-fold lower than the TFP IC_50_ measured in GM (Fig. 6I). In summary, we found a single 24-hour dose of TFP eliminated >25% of GBM cells from over half the patient lines in our cohort (*n* = 13 of 25). This effect was primarily exclusive to GBM cells exposed to CSF.

Last, we compared the effect of a single 24-hour dose of 10 μM TFP on GBM cells in GM and CSF following chemoradiotherapy with 100 μM TMZ for 7 days and 5 × 2 Gy fractions of radiation over the final 5 days of TMZ treatment (Fig. 6, K and L, and fig. S10, A, B, E, and G). We again observed a range of responses to combined treatment (fig. S10, A and B). However, the addition of TFP to cells in GM improved the response to therapy in only two cell lines (Fig. 6K). In contrast, there was a statistically significant improvement in response to TMZ and irradiation with the addition of TFP in CSF for 10 cell lines (Fig. 6K), with a mean reduction in resistance to therapy of 45% (Fig. 6L). The decrease in resistance to combined therapy in CSF was more modest for the remaining 15 cell lines yet still showed a statistically significant mean reduction of 10% (Fig. 6L). The response to treatment for each cell line in GM and CSF is summarized in fig. S10E. In summary, the addition of TFP to GBM cells treated with TMZ and irradiation improved therapeutic efficacy, an effect that was selective for cells exposed to CSF.

### Increased NUPR1 expression up-regulates its downstream transcriptional regulatory activity in GBM cells, which is altered by TFP

NUPR1 is a transcription factor that binds DNA and regulates the chromatin state and transcription of target genes by interacting with various cofactors (*44*). NUPR1 is known to up-regulate the gene expression of *ATF4*, *DDIT3*, *SPP1*, and *TFAM* (*44*–*46*). When artificially increasing the expression of NUPR1 in GBM cells cultured in GM, we observed a two-fold increase in *NUPR1* mRNA, which was accompanied by a similar ~2-fold increase in expression of NUPR1 transcriptional targets *ATF4*, *DDIT3*, *SPP1*, and *TFAM* (fig. S9C). These findings confirm that increased expression of *NUPR1* is accompanied by increased NUPR1 transcriptional activity in our patient-derived GBM model.

TFP is an established inhibitor of NUPR1 (*42*, *43*). However, to confirm the effect of TFP on NUPR1 pathways in our patient-derived GBM cell model, we examined the expression of known NUPR1 target genes in the presence or absence of the drug when artificially overexpressing NUPR1. When treated with TFP, *NUPR1* expression increased further (fig. S9D), which suggests some compensatory feedback mechanism triggered by TFP. However, in the presence of TFP, we did not observe a proportional increase in target gene expression; fold change increased expression of *ATF4*, *DDIT3*, *SPP1*, and *TFAM* was statistically less than those of *NUPR1* (fig. S9, D and E), which confirms that TFP treatment reduces the downstream transcriptional activity of NUPR1 in GBM cells.

Our results suggest that the inhibition of NUPR1 transcriptional pathways at least partially mediates the therapeutic effect of TFP on GBM cells in CSF. However, TFP is known to have other non-NUPR1–related targets (*47*). For example, it is clinically approved in the United States for treating hallucinations in schizophrenia and short-term nonpsychotic anxiety, essentially for its concurrent ability to block dopamine receptors (*48*). However, we did not detect dopamine receptor expression in our 10 GBM cell lines analyzed by scRNA-seq, indicating that the TFP killing effects on the GBM cells in CSF were independent of TFP off-target capacity to block dopamine receptors (Fig. 6H). Nevertheless, dopamine receptors are expressed in CNS neurons, especially in the midbrain, and the effect of TFP in vivo may be more complex.

### Neurotoxicity of TFP and TMZ with preclinical assays of healthy human neuronal cells

**Fig. 7.**
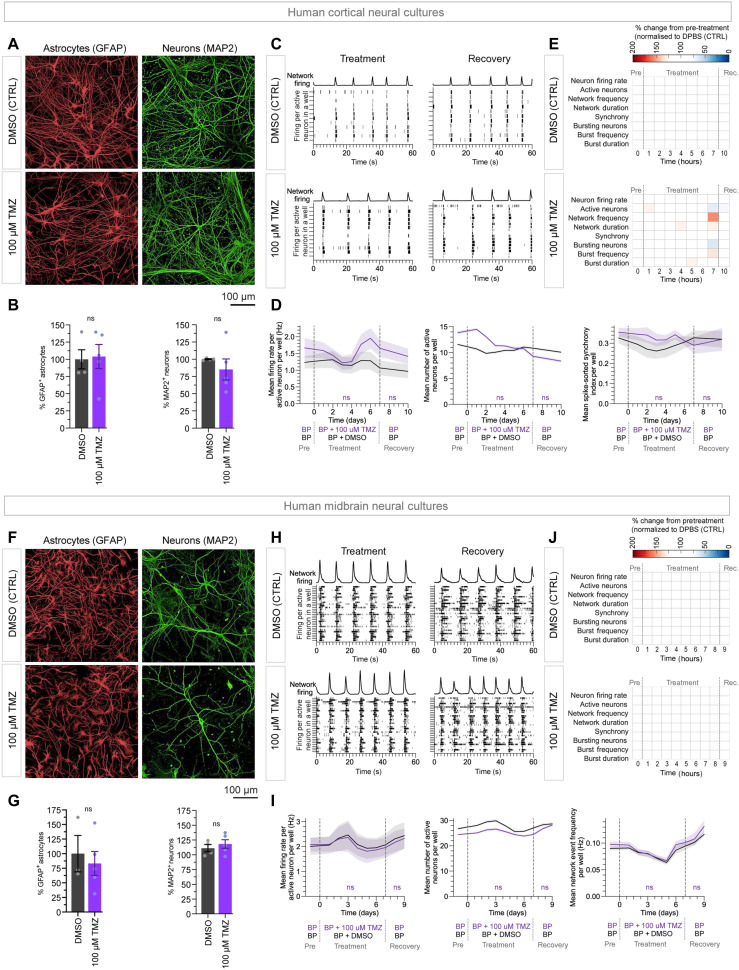
Preclinical neurotoxicity assays of TFP and TMZ on healthy human neuronal cultures. Representative images of (**A**) cortical and (**F**) midbrain neuronal cultures treated with 1:2000 DMSO or 100 μM TMZ and stained for glial fibrillary acidic protein (GFAP) (ACs), MAP2 (neurons), and with DAPI (all cells). The percentage of GFAP^+^ ACs and MAP2^+^ neurons in (**B**) cortical and (**G**) midbrain neuronal cultures with 1:2000 DMSO and 100 μM TMZ treatment. DAPI, DAPI/GFAP^+^, or DAPI/MAP2^+^ cells determined using Harmony software. Bar graphs represent the means ± SEM of six replicates. Significance was determined using two-way, unpaired Mann-Whitney tests. (**C**) Cortical and (**H**) midbrain: Raster plots depict 60 s of per-neuron firing activity in two individual wells during (day 5) and after (day 10) treatment for cortical and midbrain cultures, respectively. Each line indicates one action potential. Network firing line plots indicate synchronous firing events (arbitrary scale). (**D**) Cortical and (**I**) midbrain: Time series of single-cell spike-sorted neuronal electrophysiology data measured by multielectrode array (MEA) over 10 days. Solid lines represent the mean of data from single neurons averaged per well smoothed with a two-point moving average algorithm; the shaded area represents ±SEM. Dashed vertical lines indicate addition and removal of treatment. Six to 9 wells analyzed per condition. Area under the curve of the treatment and recovery sections were calculated in GraphPad Prism, and significance was determined by unpaired *t* test. (**E**) Cortical and (**J**) midbrain: Heatmaps represent the percent change from pretreatment for each parameter across all time points normalized to the percent change at each time point in the DMSO (CTRL). Neuronal cultures were matured for 195 days (cortical; C to E) or 100 days (midbrain; H to J) at the time of treatment. ns defined as *P* > 0.05.

**Fig. 8.**
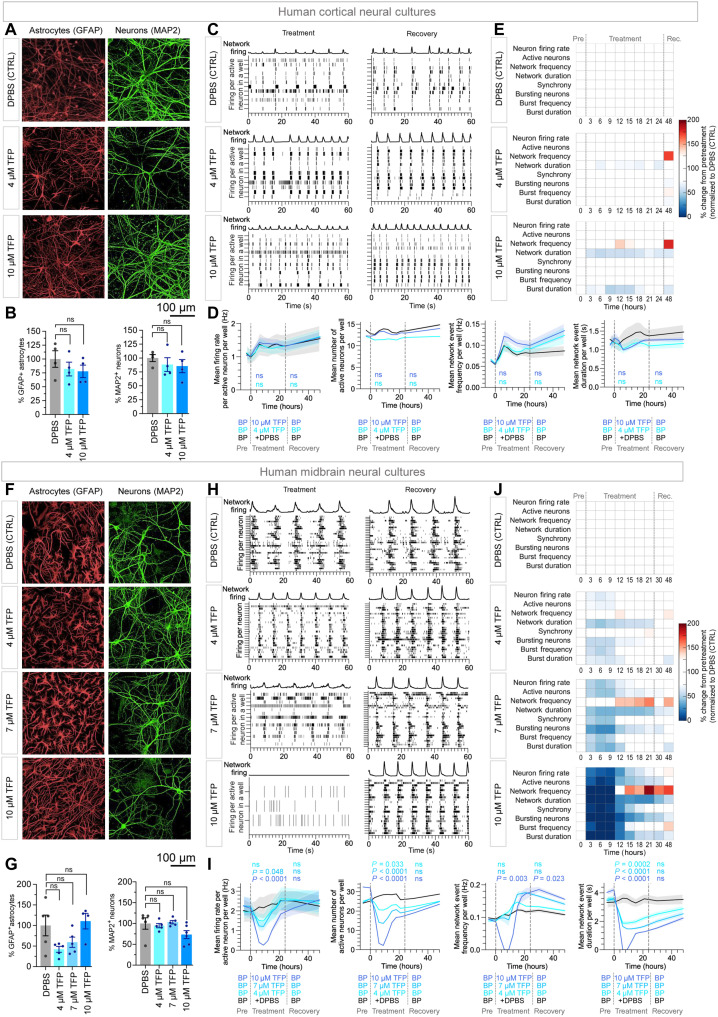
TFP has minimal effect on the survival and functionality of healthy human neuronal cultures. Representative images of (**A**) cortical neuronal cultures treated with DPBS and 4 and 10 μM TFP and (**F**) midbrain neuronal cultures treated with DPBS and 4, 7, and 10 μM TMZ. Neuronal cultures were stained for GFAP (ACs), MAP2 (neurons), and with DAPI (all cells). Percentage of GFAP^+^ ACs and MAP2^+^ neurons in (**B**) cortical and (**G**) midbrain neuronal cultures treated with DPBS or TFP. DAPI, DAPI/GFAP^+^, or DAPI/MAP2^+^ cells were determined using Harmony software. Bar graphs represent the means ± SEM of six replicates. Significance was determined using two-way, unpaired Kruskal-Wallis tests. (**C**) Cortical and (**H**) midbrain: Raster plots depict 60 s of per-neuron firing activity in two individual wells during (day 5) and after (day 10) treatment for cortical and midbrain cultures, respectively. Each line indicates one action potential. Network firing line plots indicate synchronous firing events (arbitrary scale). (**D**) Cortical and (**I**) midbrain: Time series of single-cell spike-sorted neuronal electrophysiology data measured by MEA over 10 days. Solid lines represent mean of data from single neurons averaged per well smoothed with a two-point moving average algorithm; shaded area represents ±SEM. Dashed vertical lines indicate addition and removal of treatment. Six to nine wells analyzed per condition. Area under the curve of the treatment and recovery sections were calculated in GraphPad Prism, and significance was determined by unpaired *t* test. (**E**) Cortical and (**J**) midbrain: Heatmaps represent percent change from pretreatment for each parameter across all time points normalized to percent change at each time point in the DPBS (CTRL). Neuronal cultures were matured in culture for 108 days (cortical; C to E) or 87 days (midbrain; H to J) at the time of treatment. ns defined as *P* > 0.05.

TFP and TMZ may have clinical side effects, TFP due to its dopamine receptor antagonism and TMZ due to its alkylating activity. Therefore, we investigated the sensitivity of human pluripotent stem cell–derived cortical and midbrain neurons and ACs (functionally matured for >100 days) to the similar dose treatments we used on GBM cells (0, 4, 7, and 10 μM TFP and 100 μM TMZ). Our analysis showed neither 100 μM TMZ (Fig. 7, A, B, F, and G; and figs. S11, A to C, and S12, A to C) nor 10 μM TFP (Fig. 8, A, B, F and G; and figs. S11, A, D, and E, and S12, A, D, and E) affected the viability of ACs or neurons in human cortical or midbrain neural cultures. There was no adverse effect of 100 μM TMZ on the spontaneous electrophysiological activity of cultured human cortical or midbrain neurons (Fig. 7, C to E and H to J, and fig. S13, B and D). There was also no adverse effect of 10 μM TFP on the electrophysiological activity of cultured human cortical neurons (Fig. 8, C to E, and fig. S13A). TFP was observed to reduce the electrophysiological activity of cultured human (dopaminergic) midbrain neurons at 4, 7, and 10 μM; however, the effect was transient and completely reversible at all three concentrations (Fig. 8, H to J, and fig. S13C). Overall, the TFP doses effective as a chemotherapeutic for GBM cells in CSF did not kill human neurons or ACs in vitro and only transiently modulated the electrophysiological activity of dopaminergic midbrain neuronal circuits.

## DISCUSSION

CSF critically contributes to the microenvironment of CNS cancers during tumor progression and surgery. We demonstrated CSF-induced GBM plasticity using a collection of GBM cell lines established with biopsied tumors from 25 patients. Within days of CSF exposure, we found that GBM cells were elongated, proliferated at slower rates, exited the cell cycle into G_0_ quiescent phase, and adopted more MES-like transcriptional states than under standard tissue culture conditions (GM). CSF-induced GBM cell phenotypes increased resistance to current standard treatments, TMZ and irradiation. scRNA-seq analysis revealed potential mediators of these effects, including the transcription factor NUPR1, an inhibitor of ferroptosis, whose expression was up-regulated in GBM cells in CSF and tumors of patients with worse survival outcomes. We found that the repurposed NUPR1 inhibitor, TFP, killed GBM cells in CSF more effectively than currently used treatments (TMZ and irradiation). Despite transient electrophysiological neuromodulation in healthy human midbrain neurons containing dopaminergic cells, the chemo-effective doses of TFP were not neurotoxic to healthy human cortical and midbrain neurons and ACs in vitro.

GBM tumor cells may be exposed to a CSF-rich microenvironment in several ways, before or after surgery, which may induce cellular plasticity and affect patient survival. Before surgery, malignant cells may migrate away from the tumor “core” into an otherwise healthy brain where the interstitial fluid equilibrates with CSF. Following surgery, residual tumor cells at or near the resection margin may be acutely exposed to CSF that fills the resection cavity. In addition, GBM cells may invade the lateral ventricles or extend beyond the brain parenchyma into the leptomeninges, putting them in intimate contact with CSF. Analysis of patient survival data from The Cancer Genome Atlas repository, comparing parenchymal with subependymal and leptomeningeal GBM, shows that both subependymal and leptomeningeal GBM are associated with poorer survival (fig. S14, A to D) (*23*, *49*). While both subependymal and leptomeningeal GBM are potential indicators of more advanced disease, this finding is also consistent with the hypothesis that exposure of GBM cells to CSF reduces the efficacy of chemoradiotherapy.

Just as it may affect the phenotype of GBM cells, CSF may also induce plasticity in malignant cells that metastasize to the CNS. scRNA-seq of cancer cells from the CSF of patients with leptomeningeal metastases, complemented by animal models of leptomeningeal disease, suggests metastatic cells exposed to CSF up-regulate the iron-binding protein lipocalin-2 (LCN2) and its receptor SCL22A17 to satisfy their requirement for iron (*50*). LCN2 is a transcriptional target of NUPR1, and an NUPR1-mediated increase in LCN2 has been shown to inhibit ferroptosis (*41*). Increased expression of LCN2 by metastatic cells in the CSF microenvironment thus may serve a dual role of helping to scavenge limited amounts of available iron from CSF and sequestering it within tumor cells to prevent ferroptotic cell death.

Human preclinical studies in vitro have almost exclusively been performed in glioma medium. This is supplemented with relatively high concentration of EGF and FGF2 to support the growth of glioma stem cells (*17*) but may result in a rate of tumor cell proliferation that causes an artificially high sensitivity to both TMZ and irradiation. CSF contains a different complement of growth factors, designed to maintain the physiology of the normal brain microenvironment, and is less likely to drive abnormally high cell proliferation. CSF-induced plasticity may explain the relatively poor outcome of TMZ and irradiation in improving clinical survival rates (*2*), despite efficacy in classical preclinical models (*51*). Although in vitro genetic and drug screens in glioma medium may be valid to target a subpopulation of GBM cells evolving in a tumor-dominant microenvironment, it is unlikely to capture efficacious treatments on the cells exposed to a more neuronal microenvironment. Artificial medium may bias the type of treatments progressing to clinical trials.

Our study demonstrates the need to diversify the microenvironments used in preclinical screens. However, limited access to large volumes of fresh human CSF required for preclinical assays may hinder its standardization for reproducible large-scale studies. Improving synthetic CSF substitutes may offer a practical compromise for larger-scale screens (*52*, *53*). In a subset of experiments (fig. S15), we noted that GBM cell phenotypes (cell size, proliferation, and sensitivity to TMZ and irradiation) tended to overlap better between CSF and BrainPhys medium than with standard GM. However, synthetic media have the caveat that if not perfectly optimized, they may still not accurately capture the biochemical complexity of physiological CSF. For example, a previous study found that diluting CSF 1:100 in a GM increased cell proliferation in two GBM cell lines (*54*), while we found the opposite with undiluted human CSF tested on tumor lines from 25 patients with GBM.

Another challenge of using patient-derived CSF is that its composition might fluctuate between individuals or states of the patients when it is collected (*55*). We pooled CSF samples from up to 10 individuals to minimize such bias. Future studies should examine how CSF composition fluctuations and variance may influence GBM malignancy for precision medicine. It is also worth noting that, although the collective effect of CSF on patient tumor cells was clear, not all patient cells exhibited CSF-induced plasticity and treatment sensitivity to the same extent. Future patient stratifications will be necessary to optimize personalized treatments.

Altering the patients’ CSF composition for therapeutic goals will be technically difficult or unethical. However, identifying chemotherapies that may prevent CSF-induced cell plasticity or kill CSF-induced cell types, such as TFP or other ferroptosis modulators, provides a more realistic translational solution. We identified NUPR1 as one of potential mediators of GBM cell resistance to killing by TMZ and irradiation in CSF. TMZ and irradiation are classically known to kill cancer cells by causing DNA damage, inadequate DNA repair, cell cycle arrest, apoptosis, autophagy, and necrosis (*56*, *57*). Recently, TMZ and irradiation have also been reported to induce ferroptosis (*58*, *59*), a form of cell death triggered by altered redox homeostasis, iron overload, and increased lipid peroxidation (*60*). TMZ-induced ferroptosis has been associated with increased iron uptake linked with increased expression of divalent metal transporter 1, reactive oxygen species accumulation accompanying glutathione loss, and reduced metabolism of lipid peroxides associated with reduced Glutathione Peroxidase 4 (GPX4) expression (*61*). Irradiation similarly induces reactive oxygen species and the expression of ACSL4, a lipid metabolism enzyme, resulting in elevated lipid peroxidation and ferroptosis. Irradiation also induces the expression of ferroptosis inhibitors, such as SLC7A11 and GPX4, as an adaptive response (*58*). NUPR1 is a small transcription factor and a master regulator of adaptive responses inducing ferroptosis. NUPR1-mediated induction of LCN2 expression inhibits ferroptotic cell death by reducing iron accumulation and subsequent oxidative damage (*41*). We found that CSF increased *NUPR1* gene expression in GBM cells, which has been shown to reduce ferroptosis from TMZ and irradiation (*41*, *61*). Therefore, we hypothesized that agents that inhibit NUPR1 are ideal to augment the therapeutic activity of both TMZ and irradiation in CSF. We found that TFP, an inhibitor of NUPR1, selectively killed GBM cells in CSF and could double the efficacy of current treatment combining TMZ and irradiation. TFP is clinically approved for the treatment of schizophrenia because of its dopamine receptor antagonist activity. A previous study of dopamine receptor expression in GBM reported that TFP inhibited GBM cell growth and, more recently, increased the efficacy of irradiation, both in vitro and in preclinical animal models of GBM (*62*). The authors of these studies have pursued later-generation dopamine antagonists as potential GBM therapies, advancing one to a clinical trial for recurrent GBM (*62*). Our findings demonstrate that, in addition to any therapeutic effect it may have as a dopamine receptor antagonist, TFP may also provide therapeutic benefit for GBM as an NUPR1 inhibitor.

To our knowledge, human clinical trials evaluating TFP or NUPR1 inhibitors as adjuvant therapy to treat GBM remain to be performed. Expedited translation of TFP to the clinic as an adjuvant therapy for GBM is well supported by extensive clinical experience with this drug for psychiatric disorders and nonpsychotic anxiety. TFP has been approved clinically for the treatment of schizophrenia for more than 60 years (*63*), and its side effects are well documented and manageable. Opportunities to trial TFP for GBM may be at the onset of recurrent disease, where average NUPR1 expression is higher, or alternatively perioperatively in newly diagnosed patients with GBM, when residual tumor cells at the margin of the resection cavity are acutely reexposed to CSF. If proven effective in clinical trials, then TFP will complement standard of care including surgery and chemoradiotherapy to target more specifically CSF-exposed GBM cells.

An important clinical challenge in the management of GBM is the identification of biomarkers that help predict which patients are likely to respond to treatment. Younger age, higher Karnofsky performance score and extent of surgical resection are general predictors of better overall response to the current standard of care. More specifically, low MGMT expression, associated with methylation of the MGMT gene promoter, predicts responsiveness to TMZ, and the percentage of tumor cells that are actively proliferating (%Ki67^+^ cells) correlates with radiosensitivity. We observed similar positive correlations with our panel of patient-derived GBM cell lines—those that were most sensitive to TMZ had little to no detectable MGMT expression (Fig. 2B), while there was a strong correlation between radiation sensitivity and %Ki67^+^ cells (fig. S3C).

All patient cell lines exhibited some degree of radiosensitivity, but only half of them (13 of 25) were significantly sensitive to TMZ in GM. Accordingly, we found that CSF increases the resistance of GBM cells to radiation broadly, but it increased TMZ resistance in a smaller percentage of GBMs, mostly because half of them were already TMZ-resistant in GM. In contrast, we found no patient-derived GBM lines became more sensitive to TMZ and irradiation in CSF.

In the 30% (8 of 25) most TMZ-sensitive cell lines in GM, whose resistance to TMZ increases in CSF, *MGMT* expression, which was undetectable or low in GM, did not change significantly in CSF, while *NUPR1* expression increased. This indicates additional resistance mechanisms to TMZ in CSF, possibly linked with NUPR1 transcriptional pathways and/or their reduced rate of proliferation.

Furthermore, over half of the most TFP-responsive GBM cell lines were among the most TMZ-sensitive, yet TMZ-sensitivity alone did not necessarily predict TFP sensitivity (Fig. 2B, group 1, versus Fig. 6F, group 10). However, the combination of low *MGMT* expression (Fig. 1B) and elevated *NUPR1* expression (Fig. 6B) may be a suitable biomarker to predict responsiveness to TFP in patients with GBM, and ongoing studies will investigate this. Identifying biomarkers of TFP-sensitivity will be an important prerequisite for patient selection in future clinical trial design.

Testing chemotherapies on human cells expedites translation. However, determining the chemotherapeutic doses that are effective on patient tumor cells in vitro and clinically relevant is challenging. It is also relatively easy to find drug doses killing cancer cells but harder to confirm their safety for healthy cells. We demonstrate that human induced pluripotent stem cell neuronal cultures can be used with bioelectronic assays and high-content imaging to confirm that chemo-effective doses are safe for healthy surrounding brain tissue. We identified the optimal TFP dose balancing maximum efficacy and minimum neurological side effects around 5 to 10 μM in vitro when used only for 24 hours. Dose adjustments, when delivered systemically, will be necessary. Existing clinical insights from repurposed drugs such as TFP for non-GBM neuropsychiatric patients can guide adjustment of in vivo drug regimens, to reach concentrations in the brain in vivo (or measured in CSF punctures) equivalent to the optimal levels determined in our preclinical assays.

In summary, in heterogeneous cancers such as GBM, in vitro studies that use large panels of representative cell lines maintained under translationally relevant culture conditions will likely lead to more reliable biological insights. The tumor microenvironment has an important influence on the phenotype of cancer cells, particularly the generation of heterogeneity and the resulting diversity of clinical responses. The CNS provides a unique microenvironment for cancers, and CSF is a key component. Here, we reveal the role of human CSF biochemistry in GBM plasticity and treatment sensitivity. These results expose some molecular mechanisms underlying CSF-induced plasticity in GBM cells and demonstrate that inhibiting the transcriptional regulator NUPR1 can improve the efficacy of current chemoradiotherapies for GBM in a neuronal microenvironment. By identifying trifluoroperazine as a readily translatable repurposed drug inhibitor of NUPR1, we have highlighted a clinical pathway that may improve the treatment of this currently incurable cancer.

## MATERIALS AND METHODS

### Patients with GBM

GBM tumor tissue was provided by the South Australian Neurological Tumour Bank (Flinders University) from patients undergoing surgery at Flinders Medical Centre or the Royal Adelaide Hospital. The study was approved by the human ethics committee of the South Australian Local Health Network. All patients provided informed consent. Clinical patient information is provided in table S1.

### GBM patientderived cell lines

GBM patient-derived cell lines were established in the Laboratory for Human Neurophysiology and Genetics, South Australian Health and Medical Research Institute (SAHMRI) (SANTB00423, SANTB00442, SANTB00448, SANTB00468, SANTB00469, SANTB00495, SANTB00497, SANTB00501, and SANTB00510) from fresh surgical tumor tissue or obtained from The Centre for Cancer Biology, University of South Australia (SANTB00111, SANTB00134, SANTB00159, and SANTB00216) or the Q-Cell repository of the QIMR Berghofer Medical Research Institute (BAH1, FPW1, HW1, JK2, MMK1, MN1, PB1, RKI1, RN1, SB2b, SJH1, and WK1) (*64*). Tumor tissue was either a cavitron ultrasonic surgical aspirate—where cells and tissue fragments are collected as a saline slurry—or a surgical biopsy placed immediately in cold Ringer’s solution. Following resection, samples were transported on ice without delay for processing. Tissue was cut into small pieces and dissociated using a tumor dissociation kit (human; Miltenyi Biotec, catalog no. 130-095-929) and gentleMACS dissociator (Miltenyi Biotec) according to the manufacturer’s instructions. Briefly, tumor tissue was transferred to C tubes containing the provided buffers and enzymes to dissociate the tissue enzymatically and mechanically over 1 hour at 37°C. Red blood cells were removed by brief incubation in room temperature red blood cell lysis buffer (155 mM ammonium chloride, 12 mM sodium bicarbonate, and 0.1 mM EDTA). After centrifugation [300*g* for 5 min at room temperature (RT)], samples were washed once in DMEM/F12 and then transferred in GM [DMEM/F12 medium with GlutaMAX (Thermo Fisher Scientific, catalog no. 10565018) supplemented with SM1 with vitamin A (STEMCELL Technologies, catalog no. 5711), N2A (STEMCELL Technologies, catalog no. 7152), epidermal growth factor (5 ng/ml; STEMCELL Technologies, catalog no. 78136), and FGF-basic (5 ng/ml; STEMCELL Technologies, catalog no. 78134)] to tissue culture flasks coated with reduced growth factor Matrigel (Corning, CLS356230). Matrigel was diluted 1:100 in cold DMEM/F12, added to tissue culture flasks at 1 ml/10 cm^2^, and incubated at 37°C for 1 hour in a tissue culture incubator. The entire procedure was completed in less than 2 hours. Cells were cultured at 37°C in a 5% CO_2_/95% humidified air atmosphere. Half medium changes were performed twice weekly. Cells were passaged using Accutase (STEMCELL Technologies) and cryopreserved in GM containing 10% dimethyl sulfoxide (DMSO) (Sigma-Aldrich).

### Human pluripotent stem cell–derived midbrain and cortical neuron generation

All neuron generation, culture, and sorting procedures were conducted according to previously established protocols (*52*). Briefly, NPCs were generated using human embryonic stem cells via dual SMAD inhibition neural induction and maintained in either cortical or midbrain neural progenitor medium. Midbrain neural progenitor medium was composed of DMEM/F12 + GlutaMAX supplemented with 1× SM1, 1× N2A, sonic hedgehog (200 ng/ml; PeproTech, catalog no. 100-45), FGF8b (100 ng/ml; PeproTech, catalog no. 100-25), 200 nM ascorbic acid (Sigma-Aldrich, catalog no. A4403), and laminin (1 μg/ml). Cortical neural progenitor medium consisted of DMEM/F12 + GlutaMAX plus SM1, N2A, FGF2 (10 ng/ml; STEMCELL Technologies, catalog no. 78134), 200 nM ascorbic acid, and laminin (1 μg/ml). Cultured NPCs were maintained at high densities, and medium changes were conducted every other day. Once cells reached confluency, cells were dissociated using Accutase and plated at 1.58 × 10^5^ cells/cm^2^ into Matrigel-coated plates. For neural maturation, NPCs were dissociated and seeded onto tissue culture plates coated with laminin (5 μg/ml; Thermo Fisher Scientific, catalog no. 23017015) in either cortical or midbrain neural maturation medium (NMM). NMM consisted of BrainPhys neuronal medium supplemented with specific factors to encourage midbrain or cortical fate. Midbrain NMM supplements consisted of: N2A, SM1, 200 nM ascorbic acid, 1.2 nM laminin (1 μg/ml), 20 ng of BDNF (STEMCELL Technologies, catalog no. 78133.1), 20 ng of GDNF (STEMCELL Technologies, catalog no. 78139.1), and 0.5 mM dibutyryl cyclic adenosine 5′-monophosphate (Sigma-Aldrich, catalog no. D0627). Cortical NMM supplements consisted of N2A, SM1 without vitamin A (STEMCELL Technologies, catalog no. 05731) 200 nM ascorbic acid, 1.2 nM laminin (1 μg/ml), 20 ng of BDNF, 20 ng of GDNF, IGF (STEMCELL Technologies, catalog no. 78022.1), and 0.5 mM dibutyryl cyclic adenosine 5′-monophosphate. Neurons were maintained for 14 days following differentiation with half medium changes performed every 2 to 3 days before being replated on poly-l-ornithine (10 μg/ml; Sigma-Aldrich, catalog no. P3655) and laminin (5 μg/ml)–coated tissue culture plates in either cortical or midbrain NMM containing half concentrations of growth factors. Plates were maintained at 37°C with 5% CO_2_ in standard tissue culture incubators. Neuronal cultures were functionally matured for >85 days in NMM before experiments.

### Human cerebrospinal fluid

CSF was obtained from the South Australian Cerebrospinal Fluid Biobank (Flinders University) from patients with normal pressure hydrocephalus and idiopathic intracranial hypertension. CSF collection was approved by the human ethics committee of the South Australian Local Health Network, and all patients provided informed consent. CSF was collected via lumbar puncture into 10-ml tubes and immediately stored at 4°C. After centrifuging at 2000*g* for 10 min at 4°C, cell-free CSF was stored at −80°C. Before use, CSF samples were filter-sterilized and combined in batches to reduce variability. All samples were clear and colorless. The osmolality (measured using a Fiske Micro-Osmometer, Model 210), protein concentration, and glucose concentration of each sample are reported in table S2. The glucose concentration of CSF samples was measured using a FreeStyle Optium Neo H blood glucose meter (Abbott).

### GBM morphology, proliferation, and drug screening assays

For morphology and proliferation assays, cells were seeded in 384-well CellCarrier Ultra imaging plates and precoated with reduced growth factor Matrigel (Corning, catalog no. CLS356230), at 500 to 2000 cells per well, with lower seeding densities for faster growing cell lines (*n* = 6 replicates each). For drug screening assays, cells were seeded at 2000 cells per well (*n* = 6 replicates each). Sterile water (100 μl) was added to all wells not containing cells. Cells were fixed in 4% paraformaldehyde and stained with DAPI (1:1000; Sigma-Aldrich, D9542), phalloidin-iFluor 488 (1:1000; Abcam, ab176753), CellTracker Deep Red (1:1000; Abcam, C34565), and/or anti-Ki67 (1:1000; Abcam). Briefly, GBM cells grown in 384-well CellCarrier Ultra imaging plates were washed once with 80 μl of Dulbecco’s phosphate-buffered saline (DPBS) and fixed in 4% paraformaldehyde (50 μl for 10 min at RT). Fixed cells were washed three times with 50 μl of DPBS and once with 50 μl of tris-buffered saline (TBS). CellTracker Deep Red and phalloidin-iFluor 488 were added together to cells in 50 μl of TBS per well for 1 hour at RT. DAPI was then added in 50 μl of TBS for 10 min at RT. Stained cells were washed three times with 100 μl of TBS, and 100 μl of TBS was added for imaging. For Ki67 staining, fixed cells were permeabilized in 60 μl of TBS++ (TBS, 0.2% Triton X-100, and 3% donkey serum) for 1 hour at RT. Anti-Ki67 was then added in 20 μl of TBS++ and incubated overnight at 4°C. After three washes with 80 μl of TBS, cells were incubated 30 min at RT in 40 μl of TBS++ and then 1 hour at RT in 20 μl of TBS++ containing donkey anti-rabbit Alexa Fluor 594–conjugated polyclonal antibody (1:250; Abcam, ab150068). Cells were then stained with DAPI and washed as above. All liquid transfers were performed using a Janus-automated platform (PerkinElmer).

### TMZ treatment

TMZ was dissolved in DMSO at a stock concentration of 200 mM and diluted in cell medium to the indicated concentrations, ensuring that cells were exposed to no more than 1:2000 DMSO.

### Irradiation

GBM cells in 100 μl of medium in 384-well CellCarrier Ultra imaging plates (PerkinElmer, catalog no. 6057302) received 2 Gy of ionizing radiation (RS-2000 irradiator) over 5 min, daily for 5 days. Control plates were handled identically but received no radiation.

### TFP treatment

TFP (Sigma-Aldrich, catalog no. T6062-5G) was dissolved in DPBS (Thermo Fisher Scientific, catalog no. 14190144) at a stock concentration of 104 mM and diluted to the indicated concentrations in cell medium.

### Cell imaging

Cell images were acquired with an Operetta CLS high-content imaging system (PerkinElmer) using Harmony 4.9 software (PerkinElmer). Whole wells (25 fields of view) were imaged using the 20× water confocal imaging lens, and all 25 fields of view were stitched together to generate a “Global View” image of individual wells. For all analyses, DAPI-stained nuclei were used to obtain a cell count per well. For this, DAPI-stained nuclei were identified using the “Find Nuclei” building block in Harmony 4.9 to generate an output nuclei population. The output nucleus population was then filtered based on area, distance to nearest neighbor, and distance from well border to exclude any debris, cell clumps that were detected as a single cell, or cells that were split. The number of DAPI^+^ nuclei per well was calculated using the “Define Results” building block and used to define the number of cells per well. For proliferation assays, Ki67 staining intensity within DAPI^+^ nuclei was calculated using the “Calculate Intensity Properties” building block. The number of DAPI^+^/Ki67^+^ nuclei and the percentage of Ki67^+^ nuclei were calculated using the Define Results building block. For morphology analysis, the Find Nuclei building block was first used to detect DAPI^+^ nuclei and obtain the total number object counts. The detected nuclei were used in subsequent “Find Cytoplasm” building blocks to determine the surrounding cell and cytoplasmic regions. Following detection of cell regions, area (in square micrometers), roundness, width (in micrometers), length (in micrometers), and width to length ratios were calculated for each individual cell within the well using the “Calculate Morphology Properties” building block. Similarly, mean and SD of phalloidin and CellTracker intensities were calculated using the Calculate Intensity Properties building blocks. Using the calculated intensities, a “Select Population” building block was used to exclude cells of low intensity. Similarly, cells within well borders were excluded to minimize effects of cell clumping at the border using the Select Population building block. Summary results of each well (mean, SD, median, max, min, sum, and % coefficient of variance) were calculated for each parameter (morphology and intensity) using the Define Results building block. Results were exported and analyzed using GraphPad Prism 9.

### Cytotoxicity analysis

Nuclei counts of treated replicates were normalized to paired controls with cell survival calculated as a percentage of the normalized nucleus counts.

### Neurotoxicity assay of neurons

For the assessment of neurotoxicity, human pluripotent stem cell–derived cortical or midbrain neurons were plated in 384-well CellCarrier Ultra Imaging Plates (PerkinElmer, catalog no. 6055302) at a density of 13,000 cells per well. Neuronal cultures were functionally matured for >100 days with cortical or midbrain NMM before experiments. Neurotoxicity assays were performed in parallel with multielectrode array (MEA) neuron function assays. Briefly, cells were treated with 0, 25, and 100 μM TMZ for 7 days or 0, 4, 7, and 10 μM TFP for 24 hours (*n* = 6 replicates each). Plates were incubated at 37°C with 5% CO_2_, and half-medium changes were performed every 48 hours. Wells were fixed and stained with DAPI, and cell culture supernatant was collected for simultaneous lactate dehydrogenase (LDH) cytotoxicity assays.

### Neurotoxicity staining

For neurotoxicity assays, cells were stained with DAPI, anti-chicken microtubule associated protein 2 (MAP2) (1:2000; Abcam, catalog no. ab5392), and anti-mouse glial fibrillary acidic protein (GFAP) (1:200; Abcam, catalog no. ab4648). Secondary antibody staining was performed with donkey anti-mouse Alexa Fluor 647 (1:250; catalog no. ab150111), donkey anti-chicken Alexa Fluor 488 (1:250; Stratech Scientific, catalog no. 703-545-155), and donkey anti-rabbit Alexa Fluor 568 (1:250; catalog no. AB175692). All immunofluorescence staining procedures were conducted as for GBM cell lines using the Janus-automated platform.

### LDH cytotoxicity assays

LDH cytotoxicity assays were performed using a Cytotox96 Non-Radioactive Cytotoxicity Assay Kit (Promega, catalog no. G1780). Cytotox96 reagent and positive control were prepared according to the manufacturer’s instructions. Cytotox96 reagent was added to cell culture supernatant from neurotoxicity assays and incubated at RT for 30 min. Stop solution was added following incubation, and absorbance was measured at 490 nm. Fresh medium was used as a negative control, and all samples were tested in triplicate.

### Human neuronal culture on MEA plates

Neuronal cultures were generated from NPCs in cortical or midbrain NMM as described above. After 14 days in NMM, cultures were dissociated using Accutase, strained through 40-μm filters, and resuspended in either cortical or midbrain NMM at 30,000 viable neurons/μl. A 10-μl droplet of cell suspension was added directly over the recording electrodes of each well of a 48-well Lumos MEA plate (Axion Biosystems, catalog no. M768-tMEA-48OPT), previously coated with poly-l-ornithine (10 μg/ml) and laminin (5 μg/ml). Sterile water was added to the area surrounding the wells, and the MEA plate was incubated at 37°C with 5% CO_2_ in a cell culture incubator. After 1 hour, 300 μl of either cortical or midbrain NMM was gently added to each well. Half the culture medium was exchanged with fresh medium three times per week. Neuronal cultures were functionally matured for >85 days in NMM before experiments.

### MEA analysis of neuronal function

To assess the effects of chemotherapies on neuronal function, TFP (0, 4, 7, or 10 μM) or TMZ (25 or 100 μM) was added to designated 48-well Lumos MEA plates containing 16 low-impedance 50-μm diameter microelectrodes per well (768 channels per plate) with 350-μm spacing. MEA recordings were conducted using the Maestro Pro MEA system (Axion Biosystems) at 37°C with 5% CO_2_. Recordings started 10 min after transferring a plate into the MEA. Each recording was made across a minimum of 7 min at a sampling frequency of 12.5 kHz. Recording sessions were conducted daily before and after treatment (recovery period). During the 24-hour TFP treatment, recordings were performed every hour for 6 hours, followed by recordings every 3 hours; during the 7-day TMZ treatment, recordings were taken every 1 to 2 days. Wells were washed with NMM after the end of treatment period. Voltage potentials were recorded across all channels using Version 3.6 AxIS Navigator acquisition software (Axion Biosystems), and spikes detected using an adaptive threshold set at six SDs above the mean background noise (calculated per-electrode). Spike-sorting was conducted using Plexon Offline Sorter version 4.5 (Plexon Inc.) and the in-built 3D T-Dist E-M algorithm (degrees of freedom multiplier of 10 and initial number of units set at 8). For subsequent data analysis, single-cell spike-sorted files were processed in Neural Metric Tool version 3.1.7 for active neurons (>5 spikes/min). Bursts were identified per neuron as a minimum of five spikes, and a maximum inter-spikes interval (ISI) of 100 ms. Network events were detected across all neurons with a maximum ISI of 80 ms and a minimum of 50 spikes. Raster plots, population event vectors, heatmaps, and time series were generated for selected AxIS and Plexon software data outputs using custom Python v3.8 scripts (Python Software Foundation). Raster plots were visualized as the binarized timestamps of spike-sorted neurons, which were summed at each frame and smoothed with a 500-ms rolling window for population event vectors. In time series statistical analysis, area under the curve was calculated, and values were compared by unpaired *t* test to determine significance in GraphPad Prism 9.

### Single-cell RNA sequencing

Single-cell suspensions of subconfluent GBM cells, cultured in parallel in either GM or CSF, were labeled with lipid-conjugated cell multiplexing oligonucleotides (10X Genomics) according to the manufacturer’s recommendations for cell pooling before loading onto a 10X Genomics chip. A total of 8000 cells from SANTB00134, SANTB00111, SANTB00159, and SANTB00448 and ~16,500 cells from SANTB00468, SANTB00469, SANTB00497, BAH1, HW1, and MN1 from each condition (CSF and GM) were added to the wells of an eight-channel microfluidic chip and transferred to a Chromium Controller. Single-cell RNA library preparation and sequencing were performed by the South Australian Genomics Centre and the Australian Genomics Research Facility. Single-cell pooled suspensions were loaded without delay on a GemCode Single-Cell Instrument (10X Genomics) to generate single-cell gel beads in emulsion (GEM). scRNA-seq libraries were prepared from GEMs using a 3′ CellPlex Kit Set A (10X Genomics, 1000261) and a Chromium i7 Multiplex kit (10X Genomics, 120262) according to the manufacturer’s instructions. Briefly, GEM reverse transcription incubation was performed in a 96-deep-well reaction module at 53°C for 45 min, 85°C for 5 min, and ending at 4°C. Next, GEMs were broken, and complementary DNA (cDNA) was cleaned using DynaBeads MyOne Silane Beads (Thermo Fisher Scientific, 37002D) and a SPRIselect Reagent kit (Beckman Coulter, B23318). Full-length, barcoded cDNA originating from the mRNA and from the oligonucleotide-labeled cells was polymerase chain reaction (PCR) amplified with a 96-deep-well reaction module at 98°C for 3 min; 14 cycles at 98°C for 15 s, 67°C for 20 s, and 72°C for 1 min; 1 cycle at 72°C for 1 min; and ending at 4°C. Size selection via a SPRIselect Reagent kit was used to separate the amplified cDNA molecules for 3′ gene expression library construction. Gene expression library construction to generate Illumina-ready sequencing libraries was performed after clean-up using a SPRIselect Reagent kit and enzymatic fragmentation by adding R1 (read 1 primer), P5, P7, i7 sample index, and R2 (read 2 primer sequence) via end-repair, A-tailing, adapter ligation, postligation SPRIselect clean-up/size selection, and sample index PCR. The cDNA content of prefragmentation and postsample index PCR samples was analyzed using a 2100 BioAnalyzer (Agilent). Sequencing libraries were loaded on an Illumina MiSeq, NextSeq, or Illumina NovaSeq flow cell, with sequencing settings according to the recommendations of 10X Genomics. For 3′ gene expression: read 1: 26 cycles; read 2: 98 cycles; index i7: eight cycles; index i5: no cycles, pooled in an 80:20 ratio for the combined 3′ gene expression and cell surface oligo samples, respectively.

### Single-cell data processing

Sequencing reads from 10 patient-derived GBM cell lines were demultiplexed and aligned to the GRCh38-2020-A reference using Cell Ranger 6.1.1 with default settings. Counts were generated using Cell Ranger’s “count” or “multi” function for nonmultiplexed and multiplexed samples, respectively. In total, 21 individual scRNA-seq libraries were created in this study, totaling 67,817 cells. Digital gene expression matrices were generated from sequencing data using Cell Ranger 6.1.1 (10X Genomics). The average of the mean reads per cell across all gene expression libraries was 16,383 before filtering and quality control. All downstream quality control procedures were performed using Seurat 4.1.0 in R studio. A prefiltering step was performed to keep cells expressing a minimum of 200 genes (to get rid of any empty droplets) and genes expressed in at least three cells (fig. S5A). Outlier cells were then identified on the basis of four metrics (number of reads, number of expressed genes, number of housekeeping genes, and proportion of mitochondrial genes); cells were tagged as outliers when they had >55,000 reads (>80,000 for MN1), <2000 genes (<800 for SANTB00159, <500 for SANTB00448, and <1000 for BAH1 MiSeq), <70 housekeeping genes (from a list of 98) (*31*), and >20% mitochondrial reads. Each cell line was subsampled so that “GM” and “CSF” conditions contained an equal number of cells (fig. S5, A to C).

For downstream analyses, cell lines were aggregated to create a single Seurat Object. Raw counts were normalized using default global-scaling normalization and log-transformation method (LogNormalize) in Seurat. Here, gene expression for each cell was normalized on the basis of the total expression, multiplied by a scale factor (10,000), and log-transformed. The top 2000 genes showing the highest variation between cells were detected using “FindVariableFeatures” in Seurat. Data were scaled by linear transformation. To minimize variations due to patient heterogeneity, “PatientID” was regressed during linear transformation using the “vars.to.regress” parameter in Seurat. Subsequently, the top 2000 highly variable genes were used to perform a principal components analysis with default Seurat methods. The first 25 principal components (fig. S5G) that captured most of the variability of the dataset were used to embed cells in a *k-*nearest-neighbor graph using “FindNeighbors.” The default Louvain algorithm in “FindClusters” was then used to generate cell clusters with a granularity of 0.05. UMAP plots, using the first 25 principal components, were then generated to visualize the distribution of cells.

### Calculation of S, G_2_M, quiescence, and proliferation scores

Relative S and G_2_M scores for individual cells within the dataset were calculated using gene sets provided by Tirosh *et al.* (*31*). Quiescence scores were similarly calculated using gene sets provided by Atkins *et al.* (*30*) and the “AddModuleScore” function in Seurat. The proliferation score was calculated as an average of G_1_S and G_2_M scores. Cells with a positive proliferation score (≥0) were defined as “Cycling”, while those with a negative score (<0) were defined as “NonCycling.” Similarly, cells with a quiescence score of ≥0 were termed “Quiescent,” and those with a score of <0 were termed “NonQuiescent.”

### Calculation of AC-like, MES-like, OPC-like, and NPC-like scores

Individual cells were assigned to distinct meta-modules of AC-like, MES-like, NPC-like, and OPC-like using gene lists and methods established by Neftel *et al.* (*34*). Briefly, meta-module scores were initially calculated using the AddModuleScore function. Spearman correlations of G_1_S, G_2_M, quiescent, and Neftel meta-module scores were performed to assess the relationship between GBM subtypes, cellular states, and proliferation cell-cycle phases.

### Defining AC-like, MES-like, OPC-like, and NPC-like states

To define cells as AC-like, MES-like, OPC-like, or NPC-like and visualize the distribution of cell-states, a relative meta-module *x*- or *y*-axis score was calculated for each individual cell using methods established by Neftel *et al.* (*34*). First, the maximum score between OPC/NPC and AC/MES scores was determined and assigned to “MaxScore_OPC.NPC” or “MaxScore_AC.MES.” The difference (*D*_1_) between the two scores (MaxScore_OPC.NPC − MaxScore_AC.MES) was used to define the *y* axis of the cell state plots. For cells with *D*_1_ > 0, a relative meta-module *y*-axis score was calculated as log_2_[(OPCscore − NPCscore) + 1]. For cells with *D*_1_ < 0, the relative meta-module *y*-axis score was defined as −log_2_[(ACscore − MESscore) + 1]. To plot cell distribution along the *x* axis, the maximum score between AC/OPC and MES/NPC scores was determined and assigned to “MaxScore_AC.OPC” or “MaxScore_MES.NPC.” The difference (*D*_2_) between the two scores (MaxScore_AC.OPC − MaxScore_MES.NPC) was calculated and used to determine the *x* axis of the cell-state plot. For cells with *D*_2_ < 0, the relative meta-module *x*-axis score was defined as −log_2_[(ACscore − OPCscore) + 1]. Similarly, for cells with *D*_2_ > 0, a relative meta-module *x*-axis score was calculated as log_2_[(MESscore − NPCscore) + 1]. Cell states were subsequently assigned on the basis of a cell’s relative *x*-axis and *y*-axis scores for AC-like (*x* < 0, *y* < 0), MES-like (*x* > 0, *y* < 0), NPC-like (*x* > 0, *y* > 0), and OPC-like (*x* < 0, *y* > 0).

### Differential expression analysis

The original Seurat object was randomly subsampled to contain 700 cells per condition (GM or CSF) per patient (*n* = 14,000 cells total for all 10 patients in GM or CSF). Normalization and scaling procedures were performed for the downsampled data. Differential expression analysis was then performed between CSF and GM using default parameters using Wilcoxon rank-sum test and default parameters of the “FindMarkers” function. *P* value adjustments were made using Bonferroni correction. The top 20 highly expressed GM and CSF genes (based on highest average log_2_ fold change) were visualized using the “DotPlot” function. Heatmaps and volcano plots were generated from differential expression analysis between CSF and GM for individual cell lines.

### Gene set enrichment analysis

Gene set enrichment analysis (GSEA) was performed using GSEA 4.2.2 to assess for enrichment of MES-like, MES-like 1, MES-like 2, G_1_S, G_2_M, and quiescence genes in cells cultured in GM or CSF. All GSEA was performed according to protocols established by Reimand *et al.* (*65*). Briefly, gene sets for each state (MES-like, *n* = 100; MES-like 1, *n* = 50; MES-like 2, *n* = 50; S phase, *n* = 43; G_2_M phase, *n* = 54; quiescence, *n* = 100) were loaded into the GSEA software. Differential expression analysis was performed on the downsampled Seurat object containing 7000 cells in GM/CSF. A gene rank was calculated for each gene as sign(average log_2_ fold change) * −log_10_(adjusted *P* value) giving a positive and negative score to highly expressed genes in CSF and GM, respectively. Enrichment analysis was performed using GSEA’s “pre-ranked” function with default parameters (enrichment statistic: weighted; normalization mode: meandiv; number of gene set permutations: 1000).

### Gene ontology

GO overrepresentation analysis was performed in R studio using the “enrichGO” function of clusterProfiler 4.6 (*66*). Briefly, the top 20 up- and down-regulated genes in CSF were chosen, and overrepresentation of GO biological process was performed. To minimize overlap and categorize biological processes, Louvain clustering was applied through the simplifyEnrichment 1.8 (*67*) to generate eight major clusters. All heatmaps were generated using the ComplexHeatmap 2.4 (*68*) package and chord plots using GOplot 1.0.2. New categories of biological processes were assigned on the basis of the most enriched GO processes within the clusters.

### Plasmid construction and cell transfection

*NUPR1* coding sequence was amplified by PCR from cDNA prepared from the 510 GBM cell line, using the primers NUPR1 Bam HI 5′-GAAGGATCCGCCGCCACCATGGCCACCTTCCCACCAGC-3′ (forward) and 5′-TTTGGATCCTCAGCGCCGTGCCCCTCGCTTCT-3′ (reverse) and Q5 Hot Start High-Fidelity master mix (New England Biolabs) as recommended by the manufacturer, and cloned into the Bam HI restriction site of pLV-EF1a-IRES-Puro. pLV-EF1a-IRES-Puro was a gift from T. Meyer (Addgene plasmid # 85132; http://n2t.net/addgene:85132; RRID:Addgene_85132) (*69*). The 497 GBM cell line was transfected with this plasmid, or the empty vector, using Lipofectamine 2000 (Thermo Fisher Scientific) as recommended by the manufacturer and selected using puromycin (0.5 μg/ml) for 10 days.

### Quantitative real-time PCR

PCR primers were designed using Primer3 or by Origene, with melting temperatures of approximately 60°C, to amplify unique PCR products 100 to 200 base pairs long. Total RNA was isolated from cells lysed in situ using TRIzol (Invitrogen) and purified with a Direct-zol RNA miniprep kit, using in-column deoxyribonuclease I digestion of genomic DNA. cDNA was prepared from 1 μg of total RNA using a QuantiTect Reverse Transcription kit (QIAGEN). Quantitative PCR was performed by monitoring in real time with the increase in fluorescence of SYBR Green dye (QIAGEN) with a RotorGene sequence detection system. Data were analyzed by relative quantitation using the comparative *C*_T_ method and normalized to TATA binding protein (*TBP*). Primer sequences were as follows: *NUPR1*, 5′-AGGACTTATTCCCGCTGACTGA-3′ (forward) and 5′-TGCCGTGCGTGTCTATTTATTG-3′ (reverse); activating transcription factor 4 (*ATF4*), 5′-TTCTCCAGCGACAAGGCTAAGG-3′ (forward) and 5′-CTCCAACATCCAATCTGTCCCG-3′ (reverse); DNA damage-inducible transcript 3 (*DDIT3*), 5′- GGTATGAGGACCTGCAAGAGGT-3′ (forward) and 5′-CTTGTGACCTCTGCTGGTTCTG-3′ (reverse); secreted phosphoprotein 1 (*SPP1*), 5′-CGAGGTGATAGTGTGGTTTATGG-3′ (forward) and 5′-GCACCATTCAACTCCTCGCTTTC-3′ (reverse); transcription factor A, mitochondrial (*TFAM*), 5′- GTGGTTTTCATCTGTCTTGGCAAG-3′ (forward) and 5′-TTCCCTCCAACGCTGGGCAATT-3′ (reverse); *TBP*, 5′-CACGCCAGCTTCGGAGAGTT-3′ (forward) and 5′-ATCAGTGCCGTGGTTCGTGG-3′ (reverse).

### Statistics

All statistical analyses were performed using GraphPad Prism 9. SEM was reported unless otherwise stated. Significance was assessed using two-tailed, nonparametric, Mann-Whitney, or Wilcoxon matched-pairs sum-rank tests. For dose responses, significance was evaluated using two-way analysis of variance (ANOVA). Curve fits were assessed using nonlinear, least-squares regression analysis of [inhibitor] versus normalized slope and extra-sum-of-squares *F* test. The significance level (α) was determined as *P* < 0.05.

### Materials

Key materials and reagents used in this work are summarized in table S3.
