# Supplementary material for: Human cerebrospinal fluid affects chemoradiotherapy sensitivities in tumor cells from patients with glioblastoma
Source: Sci Adv. 2023 Oct 25;9(43):eadf1332. doi: 10.1126/sciadv.adf1332 (PMC10599627; doi:10.1126/sciadv.adf1332)
Supplement: Supplementary file 1 — Figs. S1 to S15 Tables S1 to S3 [file sciadv.adf1332_sm.pdf]

Science Advances
AAAS

Supplementary Materials for

Human cerebrospinal fluid affects chemoradiotherapy sensitivities in tumor cells from patients with glioblastoma

Brett W. Stringer *et al.*

Corresponding author: Cedric Bardy, cedric.bardy@sahmri.com

Sci. Adv. **9**, eadf1332 (2023)
DOI: 10.1126/sciadv.adf1332

This PDF file includes:

Figs. S1 to S15
Tables S1 to S3

Supplementary Fig. 1

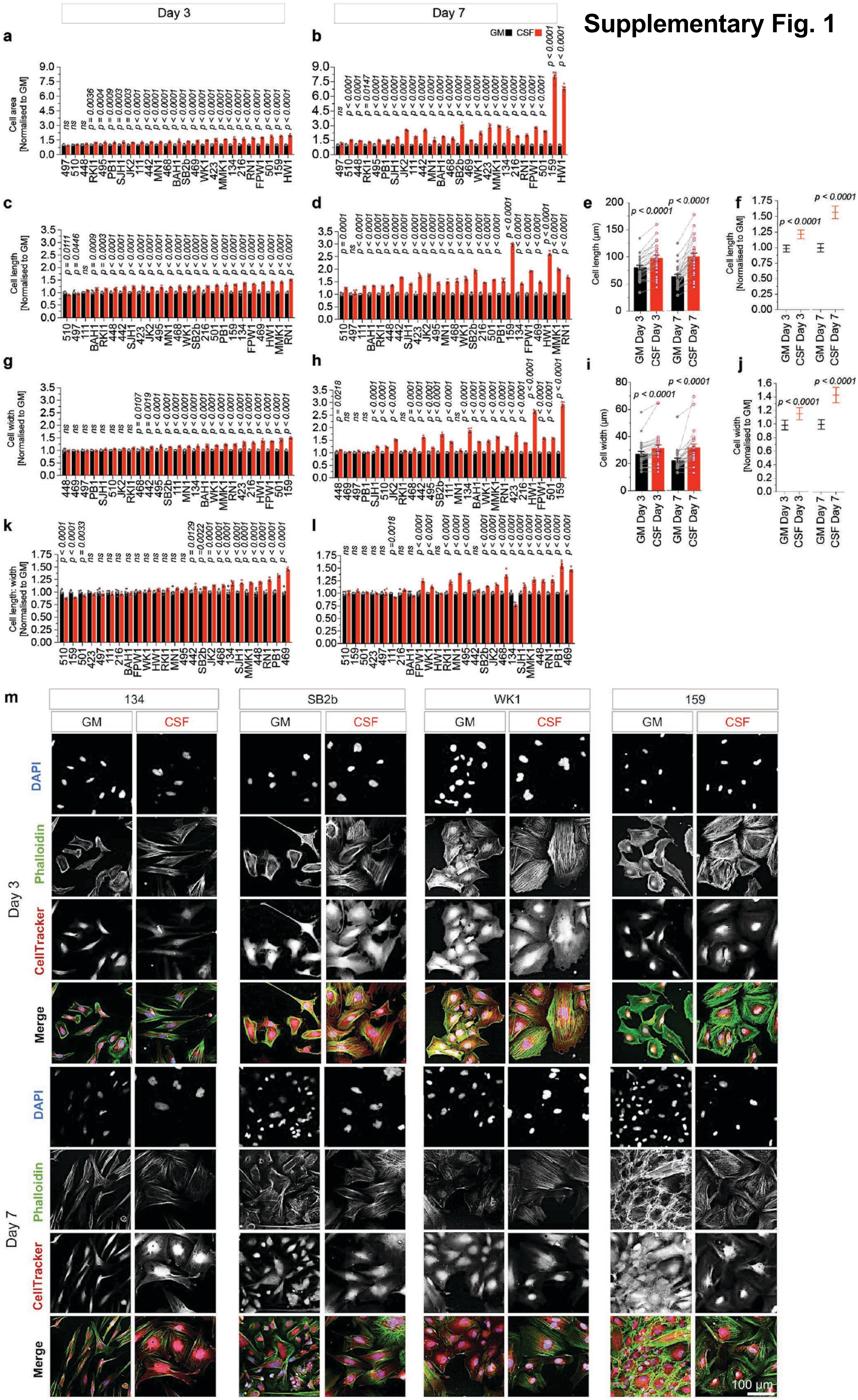

Supplementary Fig. 1 | GBM cells are larger and elongated in CSF. a-l, Morphology analysis of GBM cells from 25 patient biopsies cultured in CSF or standard glioma medium (GM) for 3- or 7-days. **a**,**b**, Cell area, **c**,**d**, cell length, **g**,**h**, cell width and **k**,**l**, cell length:width determined using Harmony™ software analysis of CellTracker™ Deep Red-stained cells (n>5000 per cell line) normalised to GM. Individual data points represent six replicates of each GBM cell line. Bar graphs represent the mean +/- SEM. Significance was determined using two-way ANOVA. Change in cell length (**e**) and cell width (**i**) of patient-derived cell cultures. Bar graphs represent the mean +/- SEM of 25 GBM cell lines. Significance was determined using two-way, paired Wilcoxon tests. **f**, Cell length and **j**, cell width normalised to GM. Graphs represent the mean +/- SEM for 25 GBM cell lines. Significance was determined using two-way, paired Wilcoxon tests. **m**, Representative images of GBM cells cultured in GM and after 3- and 7-days of exposure to CSF. Cells are stained with DAPI, phalloidin iFluor™ 488 and CellTracker™ Deep Red. ns defined as $p > 0.05$.

Supplementary Fig. 2

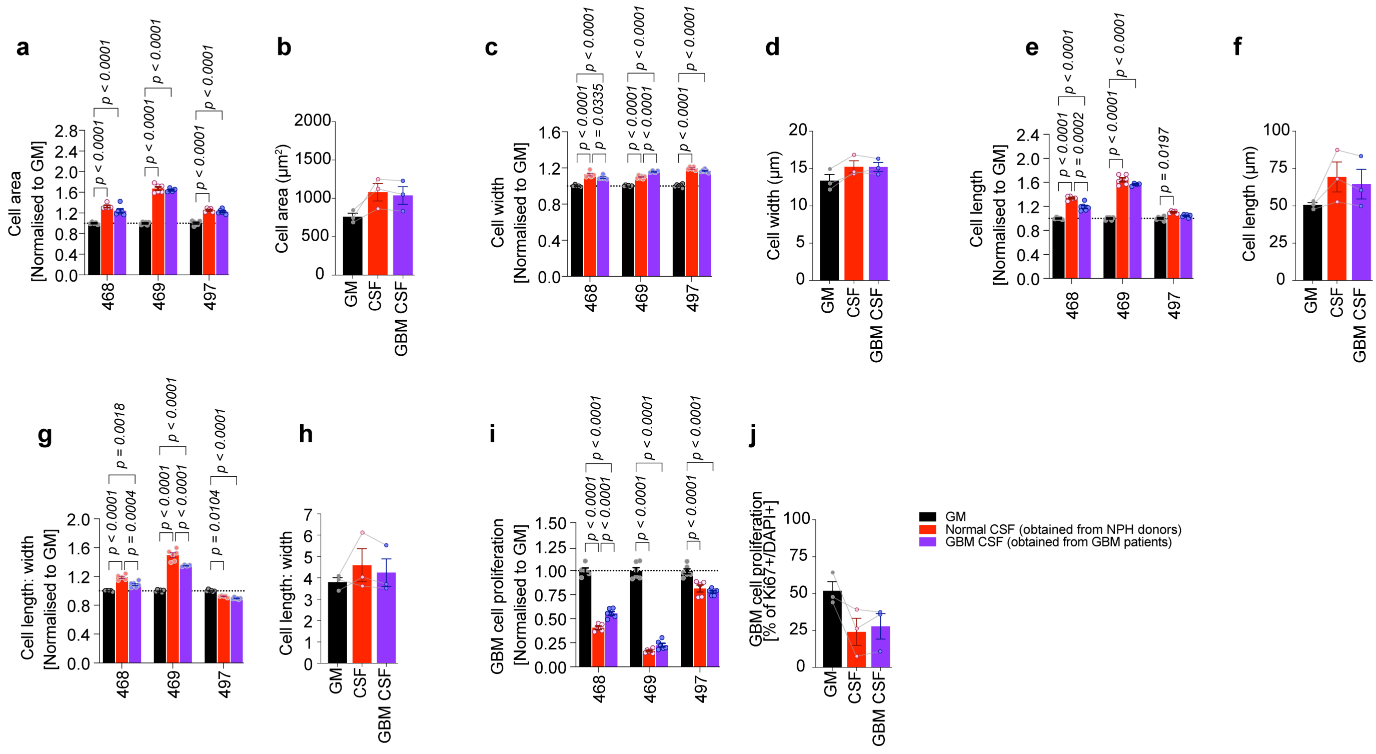

Supplementary Fig. 2 | GBM cell phenotypes were similar in normal CSF and CSF from GBM patients. a-f, Morphology analysis of patient-derived GBM cells cultured in standard glioma medium (GM), healthy, human CSF or GBM patient CSF for 4-days. Cell area, width and length of three patient-derived GBM cell lines determined using Harmony software analysis of CellTracker™ Deep Red-stained cells. **a,c,e,g** Grouped bar graphs demonstrate fold change of cell area, width, length and length: width normalised to GM controls, respectively. Individual data points represent the mean +/- SEM of six replicates. Significance was determined using two-way analysis of variance. **b,d,f,h,** Paired bar-graphs showing change in cell area, width, length and length: width, respectively. Individual dot points represent the mean of six replicates for each cell line. Bar graphs represent mean +/- SEM of three GBM cell lines. **i-j,** Proliferation analysis of patient-derived GBM cells cultured in GM, healthy, human CSF or GBM patient CSF for 4-days. Percentage of proliferating cells determined using Harmony software analysis of Ki67+ and DAPI+ cells. **i,** Grouped bar graphs demonstrate fold change of the percentage of proliferating cells normalised to GM controls. Individual data points represent the mean +/- SEM of six replicates. Significance was determined using two-way analysis of variance. **j,** Paired bar-graphs showing differences in the percentage of proliferating cells. Individual dot points represent the mean of six replicates for each cell line. Bar graphs represent mean +/- SEM of three GBM cell lines. ns defined as $p > 0.05$ and not included in graphs.

Supplementary Fig. 3

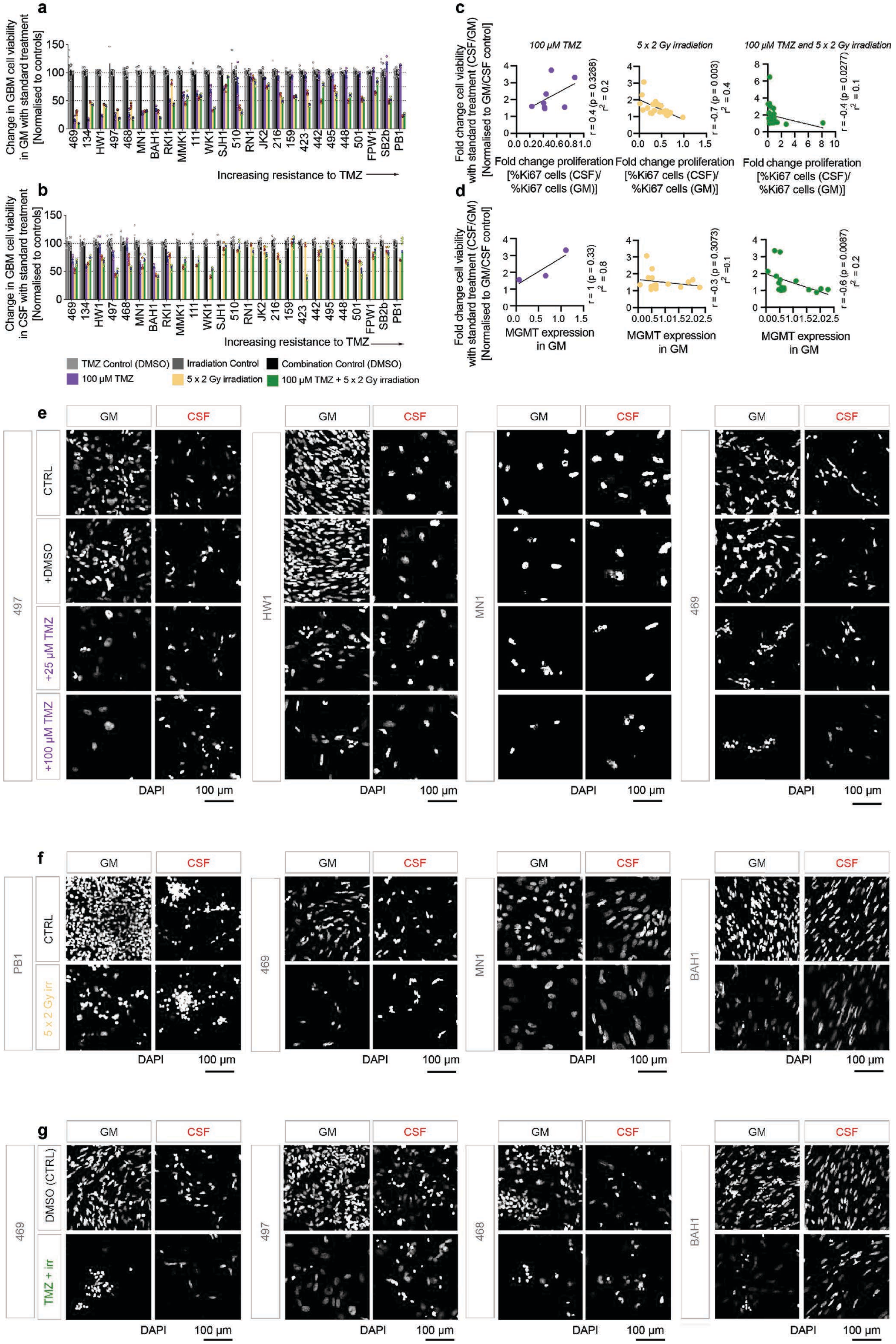

Supplementary Fig. 3 | Patient GBM cell lines display heterogeneous responses to standard therapeutics. a-g, Cytotoxicity analysis of patient-derived GBM cells cultured in CSF or GM for 3 days prior to treatment. Responsiveness of 25 patient- derived GBM cell lines cultured in **a,** GM or **b,** CSF for 3 days and exposed to 1:2000 DMSO, 100 µM temozolomide (TMZ) for 7 days, 5 fractions of 2 Gy irradiation for 5 days or 5 fractions of 2 Gy irradiation and 100 µM TMZ for 7 days. DAPI-stained cells counted using Harmony™ software. Individual data points represent six replicates for each GBM cell line. Bar graphs represent the mean +/- SEM. Significance determined using two-way ANOVA. Correlation graphs of **c,** Ki67 expression and **d,** MGMT mRNA expression against cell survival following standard treatment. **c,** Fold change of Ki67 expression in CSF compared to GM calculated using untreated controls. Fold change of cell survival in CSF compared to GM calculated by normalising treated samples to untreated controls. Correlation calculated using Spearman's R and line of best fit generated using simple linear regression. Representative images of cell lines **e,** untreated or treated with 1:2000 DMSO control, 25 µM and 100 µM TMZ for 7-days; **f,** untreated or treated with 5 fractions of 2 Gy irradiation over 5-days; **g,** treated with 1:2000 DMSO or 5 fractions of 2 Gy irradiation and 100 µM TMZ for 5- and 7-days, respectively. Cells are stained with DAPI. ns defined as $p > 0.05$.

Supplementary Fig. 4

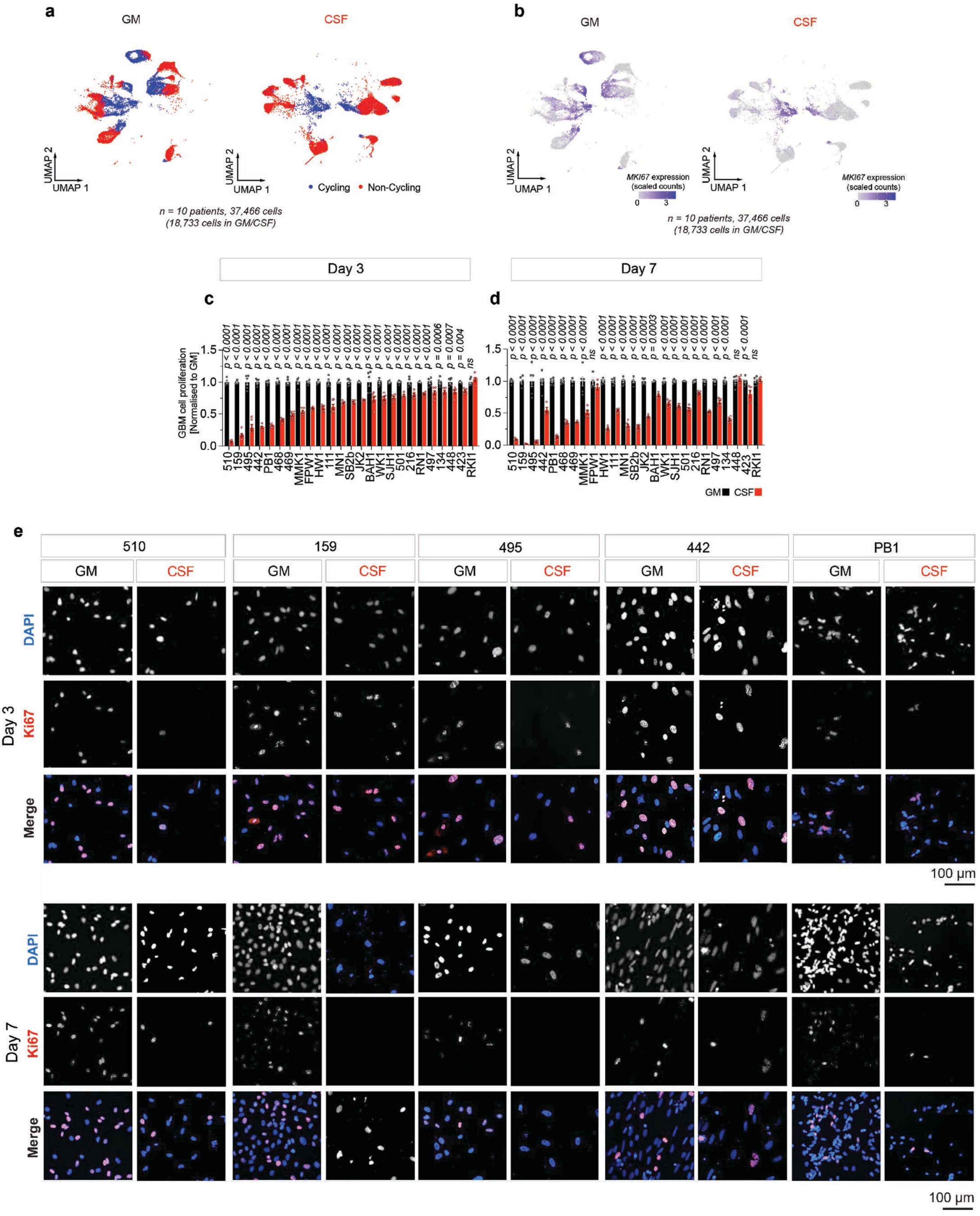

Supplementary Fig. 4 | GBM cells become less proliferative and more quiescent in CSF. a-e, Proliferation analysis of patient-derived GBM cells cultured in CSF or GM for 3- and 7-days. Uniform manifold approximation and projection (UMAP) plots showing **a**, distribution of cycling and non-cycling cells and **b**, expression of *MKI67* across 10 patient-derived GBM cell lines (n = 18,733 cells in GM/CSF). **c,** Percentage of proliferating cells after 3- and 7-days exposure to CSF determined using Harmony™ software analysis of cells expressing Ki67 and DAPI-stained cells. Individual data points represent six replicates for each GBM cell line. Bar graphs represent the mean +/- SEM. Significance determined using two-way ANOVA. **e**, Representative images of GBM cells cultured in GM and after 3- and 7-days of exposure to CSF. Cells are stained with DAPI and for Ki67.

Supplementary Fig. 5

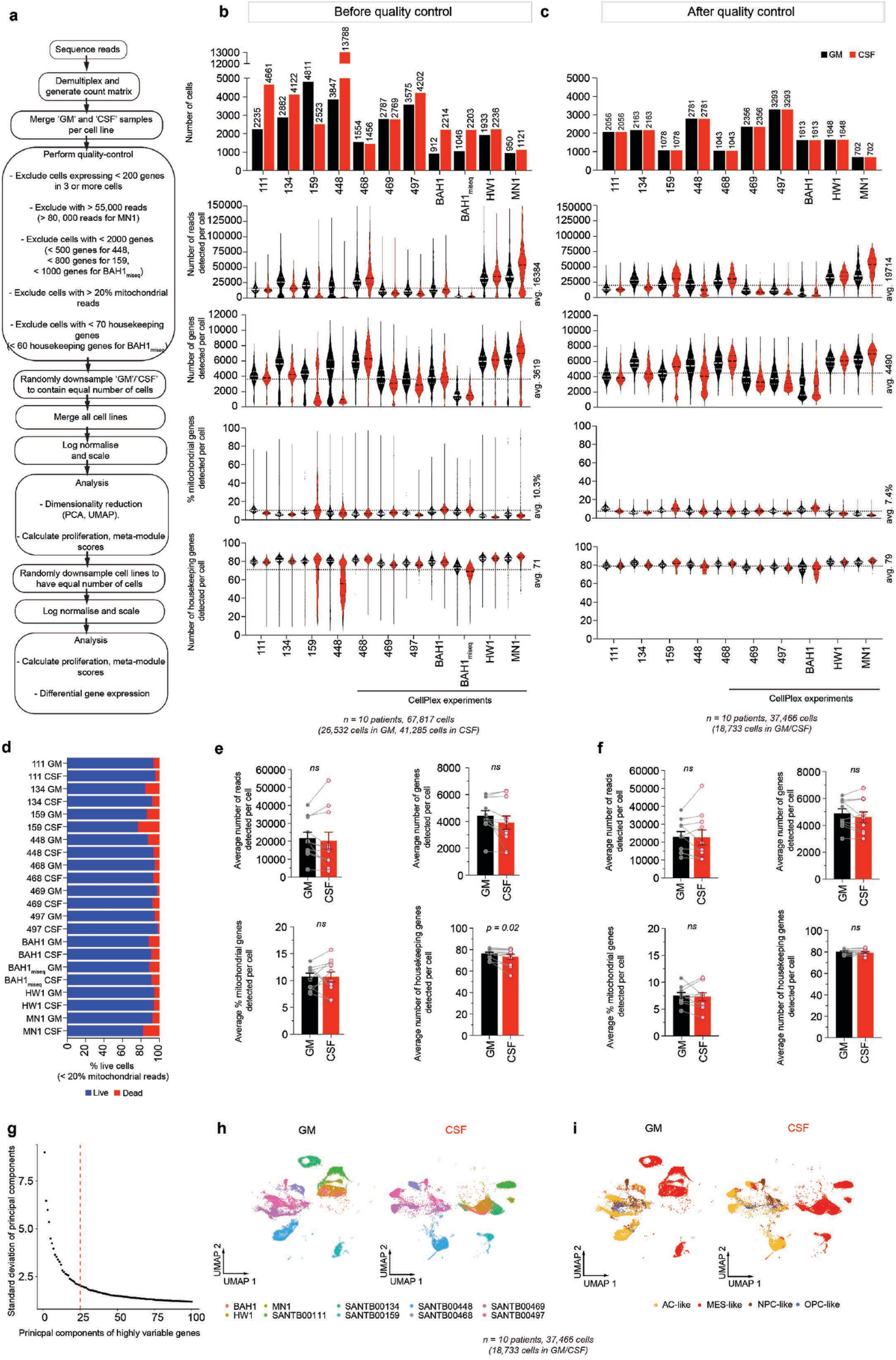

Supplementary Fig. 5 | Quality control of single-cell RNA sequencing data. a, Flow chart of workflow for single-cell RNA sequencing analysis. **b**,**c**, Bar charts and violin plots show the number of cells, reads, genes, percentage mitochondrial genes and housekeeping genes detected in GM and CSF for each individual cell line prior to filtering (**b**) and post-filtering (**c**). To increase the number of cells, novaseq and miseq sequencing runs of BAH1 were filtered independently and combined post-filtering as 'BAH1'. **d**, Percentage of live and dead cells for each individual cell line. Live and dead cells were determined as the number of cells expressing < 20% and >= 20% mitochondrial genes, respectively. **e**,**f**, Paired graphs show the average number of reads, genes, percentage mitochondrial genes and housekeeping genes detected in GM and CSF before (**e**) and after (**f**) filtering. Paired points represent individual cell lines. Error bars represent the mean +/- sem of the 10 patient-derived cell lines. Significance was determined using two-way, paired Wilcoxon tests. **g**, Elbow plot showing the standard deviation of 100 principal components calculated using the top 2000 variable genes in the dataset. Dotted red line represents the number of principal components (n = 25) used to calculate shared nearest neighbour distances, cluster cells and generate UMAP plots. **h**, UMAP plot shows the distribution of cells from 10 patient-derived cell lines according to cell-state (UMAP 1) and cell-line of origin (UMAP 2).

Supplementary Fig. 6

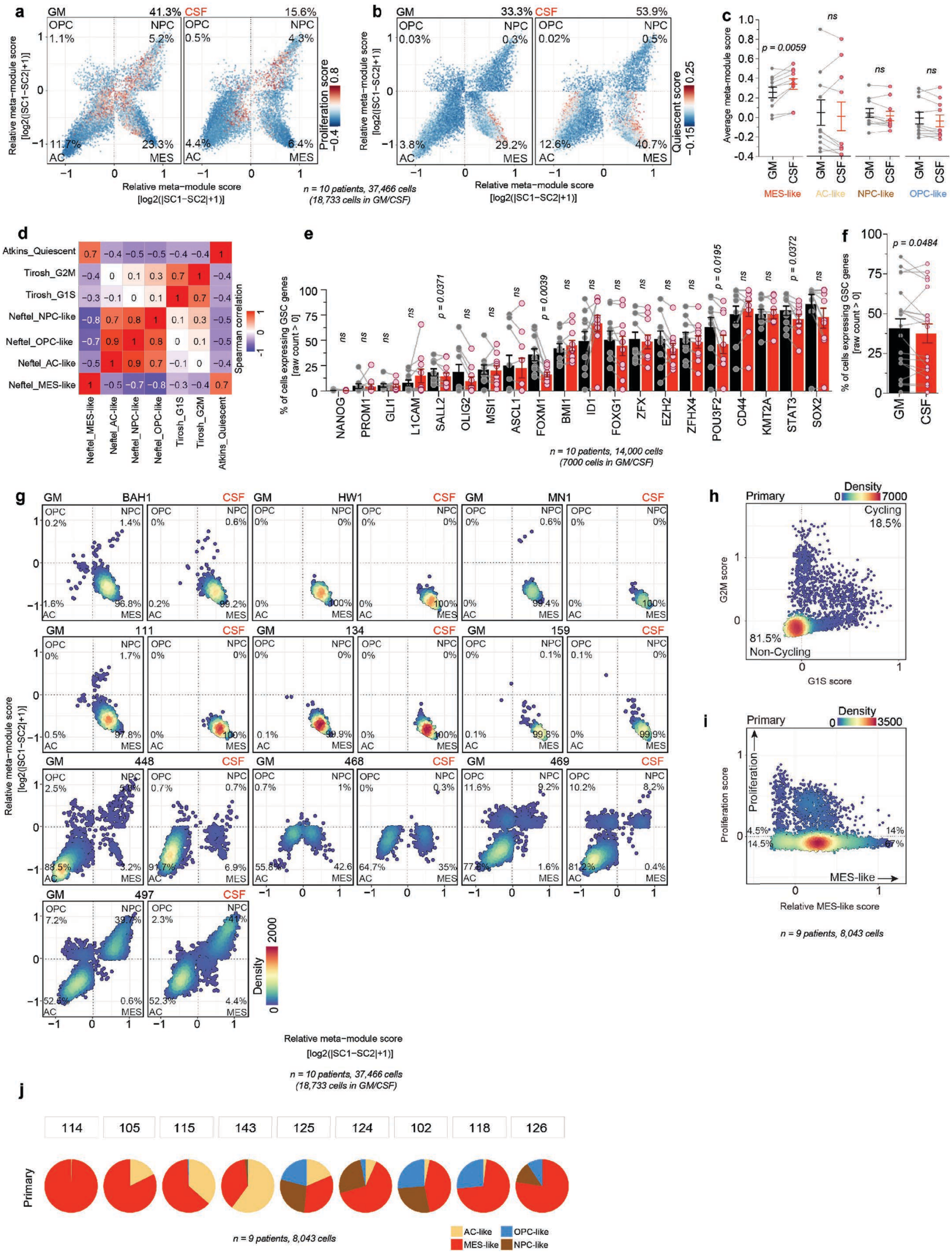

Supplementary Fig. 6 | GBM cells shift towards a mesenchymal-like state in CSF. a-e, Single-cell RNA sequencing analysis of 10 patient-derived GBM cell lines cultured in GM or CSF for 3 days (n = 18,733 cells GM/CSF). Cell-state scatter plots showing proportion of **a,** proliferating and **b,** quiescent cells for each cell-state in GM or CSF. Colour scale shows **a,** proliferation and **b,** quiescent score. Proliferation and quiescent scores determined using gene lists by Tirosh et al, 2016 (Ref: 31) and Atkins et al, 2019 (Ref: 30). **c,** Average MES-, AC-, NPC- and OPC-like scores per cell line. Error bars indicate the mean +/- SEM. Significance determined using paired, two-way Wilcoxon tests. **d,** Spearman correlation of cell state (AC-, MES-, NPC-, OPC-like), G1S, G2M and quiescent scores. **e,** Percentage of cells expressing 20 reported GBM stem-cell (GSC) genes, obtained from Lathia et al, 2015 (Ref: 38), in GM and CSF. Paired points represent 10 sequenced patient-derived GBM cell lines. Bar graphs represent mean +/- SEM. Significance determined using paired, two-way Wilcoxon tests. **f,** Percentage of cells expressing recognised GSC markers in GM and CSF. Individual points represent the percentage of cells, in each GBM cell line, expressing GSC genes in GM and CSF. Bar graphs represent mean +/- SEM. Significance determined using paired, two-way Wilcoxon tests. **g,** Density plots showing the distribution of MES-, AC-, NPC- and OPC-like cells per cell line in GM and CSF. **h,** Scatter plot represents the percentage of cycling (G1S or G2M > 0) and non-cycling cells (G1S and G2M < 0) in nine primary GBM tumours (n = 8,043 cells). G1S and G2M scores calculated using gene lists by Tirosh et al, 2016 (Ref: 30). **i,** Scatter plot represents the percentage of proliferating and MES-like cells in primary GBM tumours. **j,** Proportion of MES-like, AC-like, NPC-like and OPC-like cells in patient tumours. Normalised TPM counts were provided by Neftel et al, 2019 (34). ns defined as p > 0.05.

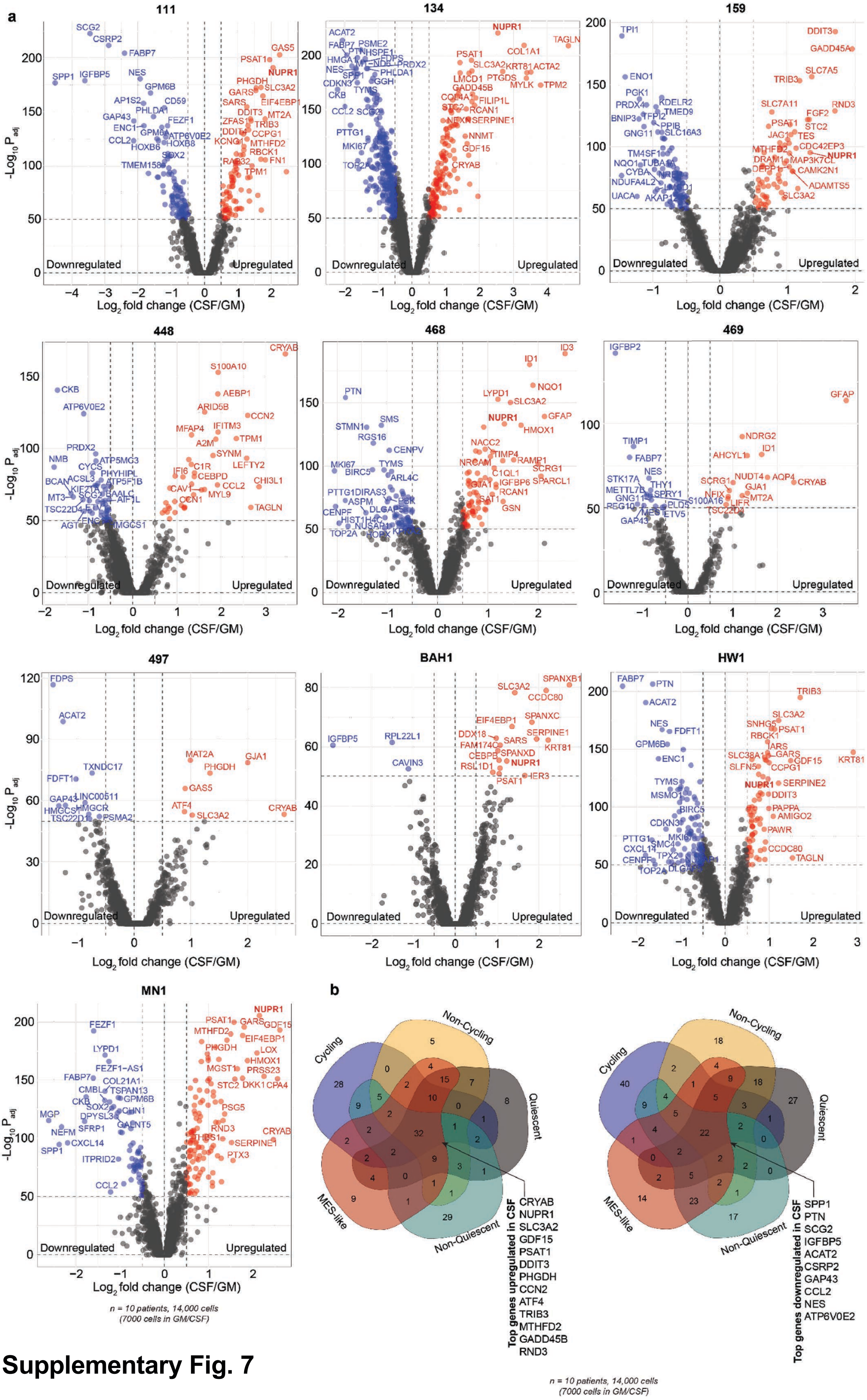

Supplementary Fig. 7

Supplementary Fig. 7 | NUPR1 and other potential therapeutic targets are upregulated upon exposure to CSF. a,**b**, Differential gene expression analysis for patient-derived GBM cell lines cultured in GM or CSF for 3-days (n = 1400 cells per patient, n = 700 cells in GM/CSF). **a**, Volcano plot showing genes that are differentially expressed (in red: $-\log10$(adjusted P) > 50, log2(FC) > 0.5; in blue: $-\log10$(adjusted P) > 50, log2(FC) < -0.5) between GBM cells in CSF relative to GM. P value adjustment was performed using Bonferroni correction. The top 20 relatively upregulated (red) and downregulated (blue) genes are labelled. **b,** Venn diagrams show top genes relatively upregulated and downregulated in CSF compared to GM. Differential gene expression analysis for CSF versus GM was performed for cycling, non-cycling, quiescent, non-quiescent and MES-like cells. Gene overlap and venn diagrams were generated using https://bioinformatics.psb.ugent.be/webtools/Venn/. Venn diagrams show the top genes upregulated and downregulated in CSF regardless of cell-cycle phase or cell-state. Data shown is for the merged cell set, randomly downsampled to 1400 cells per cell line (n = 700 GM/CSF).

Supplementary Fig. 8

a

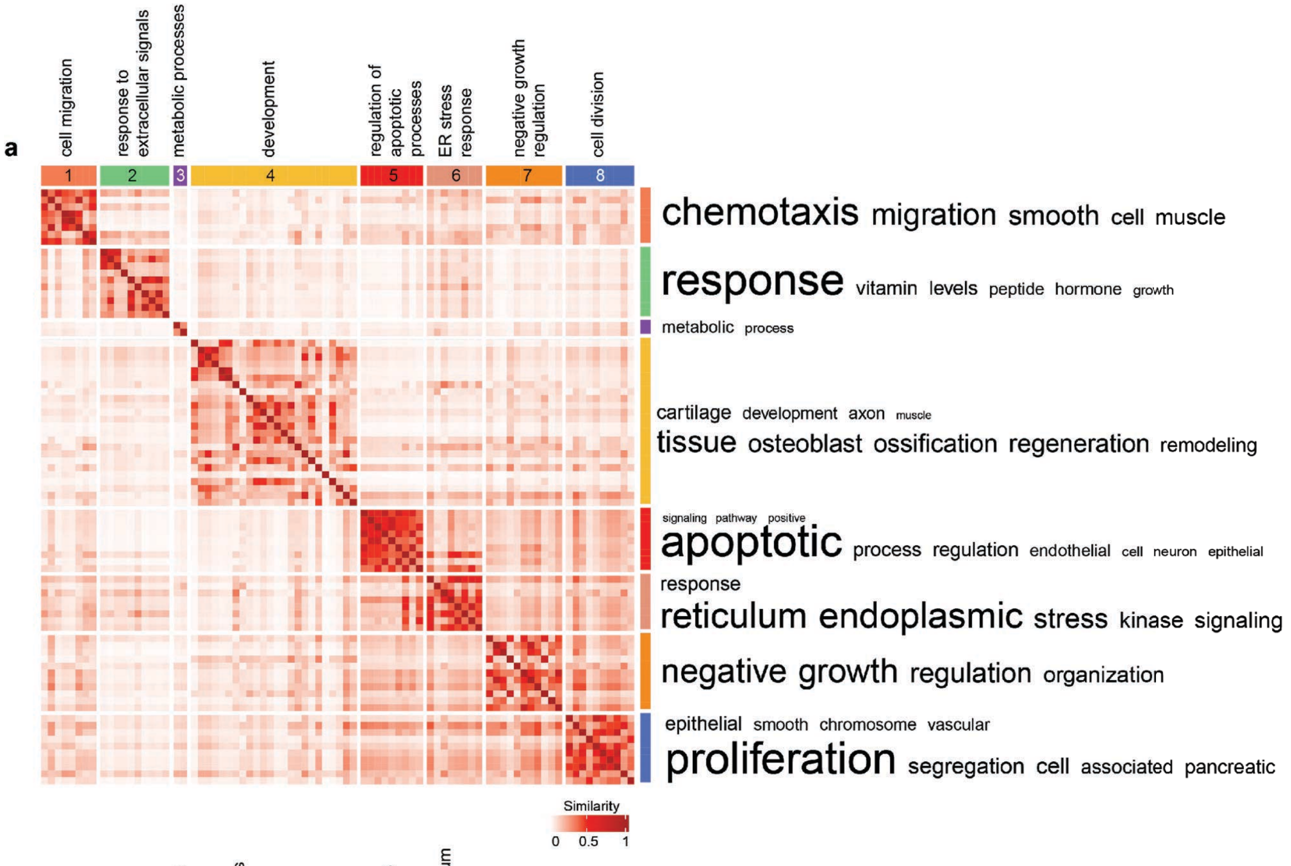

b

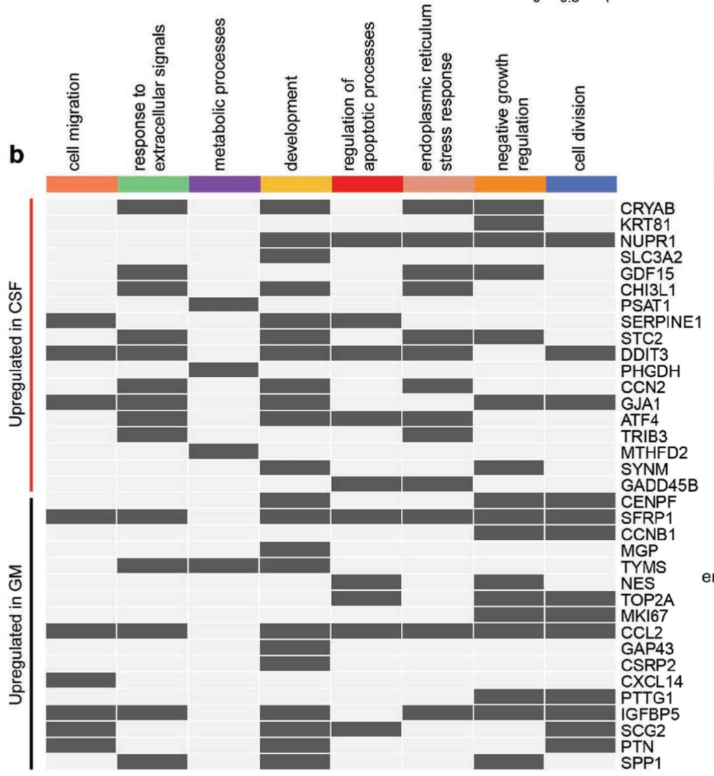

c

Description	GO Terms			
cell migration	GO:0030336	GO:0033280	GO:0046653	GO:0048771
	GO:0043523	GO:1990440	GO:2001251	GO:0051983
response to extracellular signals	GO:0033273	GO:0033280	GO:0031667	GO:0036293
	GO:1901652	GO:0044344	GO:0060416	GO:0032355
	GO:0043434	GO:0070555		
metabolic processes	GO:0006563	GO:0046653		
development	GO:0048771	GO:0001503	GO:0046849	GO:0007565
	GO:0007566	GO:0031103	GO:0032757	GO:0007568
	GO:0031214	GO:0001502	GO:0031099	GO:0051216
	GO:0001525	GO:0070167	GO:0048679	GO:0001649
	GO:0060537	GO:0030278	GO:0006816	GO:0022612
	GO:0003012	GO:0045667	GO:0044060	
regulation of apoptotic processes	GO:0043523	GO:0043065	GO:2000351	GO:0043525
	GO:1904036	GO:2001233	GO:2000352	GO:2001235
endoplasmic reticulum stress response	GO:1990440	GO:0034976	GO:0070059	GO:0051896
	GO:0030968	GO:0051897	GO:0051403	
negative growth regulation	GO:2001251	GO:0033046	GO:0040015	GO:0045104
	GO:0030308	GO:0010639	GO:0045786	GO:0048640
	GO:0007088	GO:0001558		
cell division	GO:0051983	GO:0050680	GO:1990874	GO:0050673
	GO:0061469	GO:0048145	GO:0050678	GO:0007059
	GO:1904707			

Supplementary Fig. 8 | NUPR1 and other potential therapeutic targets are upregulated upon exposure to CSF. a-c, Gene ontology (GO) over-representation analysis was performed for the top 40 differentially expressed genes in both CSF and GM. **a**, Heatmap generated following louvain clustering of significantly enriched GO biological process terms (p-adjusted < 0.05) show eight major clusters. Word cloud on the right of the heatmap displays the most frequent GO term descriptions. Similarity matrix was generated using simplifyEnrichment 1.8 and heatmap generated using ComplexHeatmap 1.8. Five of the top 40 differentially expressed genes did not belong to significantly enriched GO terms. **b,** Top 35 differentially expressed genes in GM and CSF and their distribution across the eight clusters. **c,** GO terms belonging to each new category.

Supplementary Fig. 9

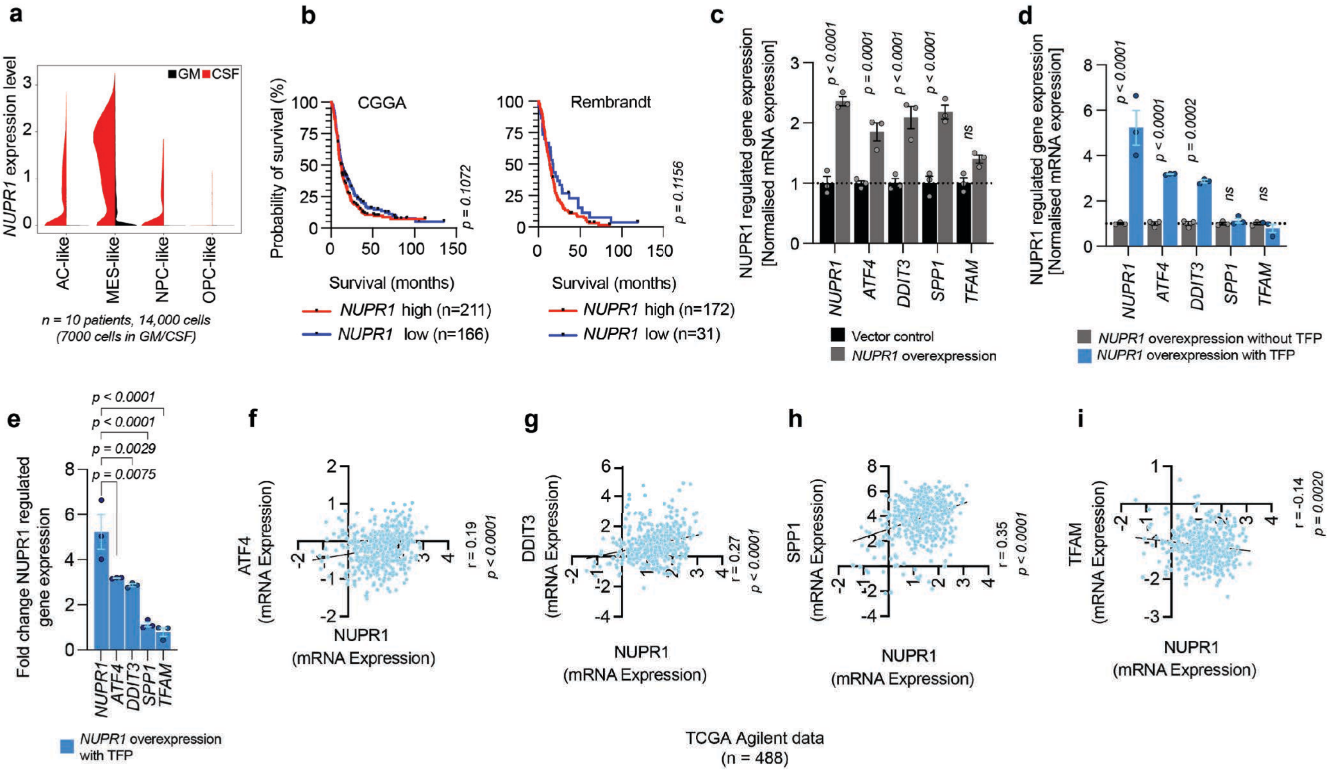

Supplementary Fig. 9 | Targeting NUPR1 with the repurposed, antipsychotic trifluoperazine reduces cell survival in CSF. a, Violin plot showing *NUPR1* expression in each of the four transcriptional cell states (n = 7,000 cells in GM/CSF). **b,** Kaplan-Meier survival curves showing the association between GBM patient overall survival and GBM *NUPR1*. Data is from the CGGA or Rembrandt GBM datasets downloaded from GlioVis. ‘NUPR1 high’ and ‘NUPR1 low’ divisions of the datasets were done using the optimum cutoff. **c-e,** Quantitative polymerase chain reaction measurement of *NUPR1* and *NUPR1*-regulated genes, *ATF4, DDIT3, SPP1* and *TFAM* in SANTB00497 stably expressing *NUPR1* or transfected with vector control and cultured in GM or GM containing 10 µM TFP for 18 hours. **c,** Expression of *NUPR1, ATF4, DDIT3, SPP1* and *TFAM* in SANTB00497 lines stably expressing *NUPR1* normalised to vector control. **d,** Expression of *NUPR1, ATF4, DDIT3, SPP1* and *TFAM* in SANTB00497 lines stably expressing *NUPR1* treated with 10 µM TFP normalised to untreated control. **e,** Comparison of fold-change between *NUPR1* and *NUPR1*-regulated genes, *ATF4, DDIT3, SPP1* and *TFAM* in SANTB00497 treated with 10 µM TFP. Gene expression was determined relative to TBP. Bar graphs represent the mean +/- SEM of triplicate measurements. **f-i,** Correlation of *NUPR1* and *NUPR1*-regulated genes, *ATF4, DDIT3, SPP1* and *TFAM.* Data is from the TCGA Agilent GBM dataset downloaded from the GlioVis web application for data visualisation and analysis. Significance of correlation was determined using Spearman’s R and line of best fit was generated through simple linear regression.

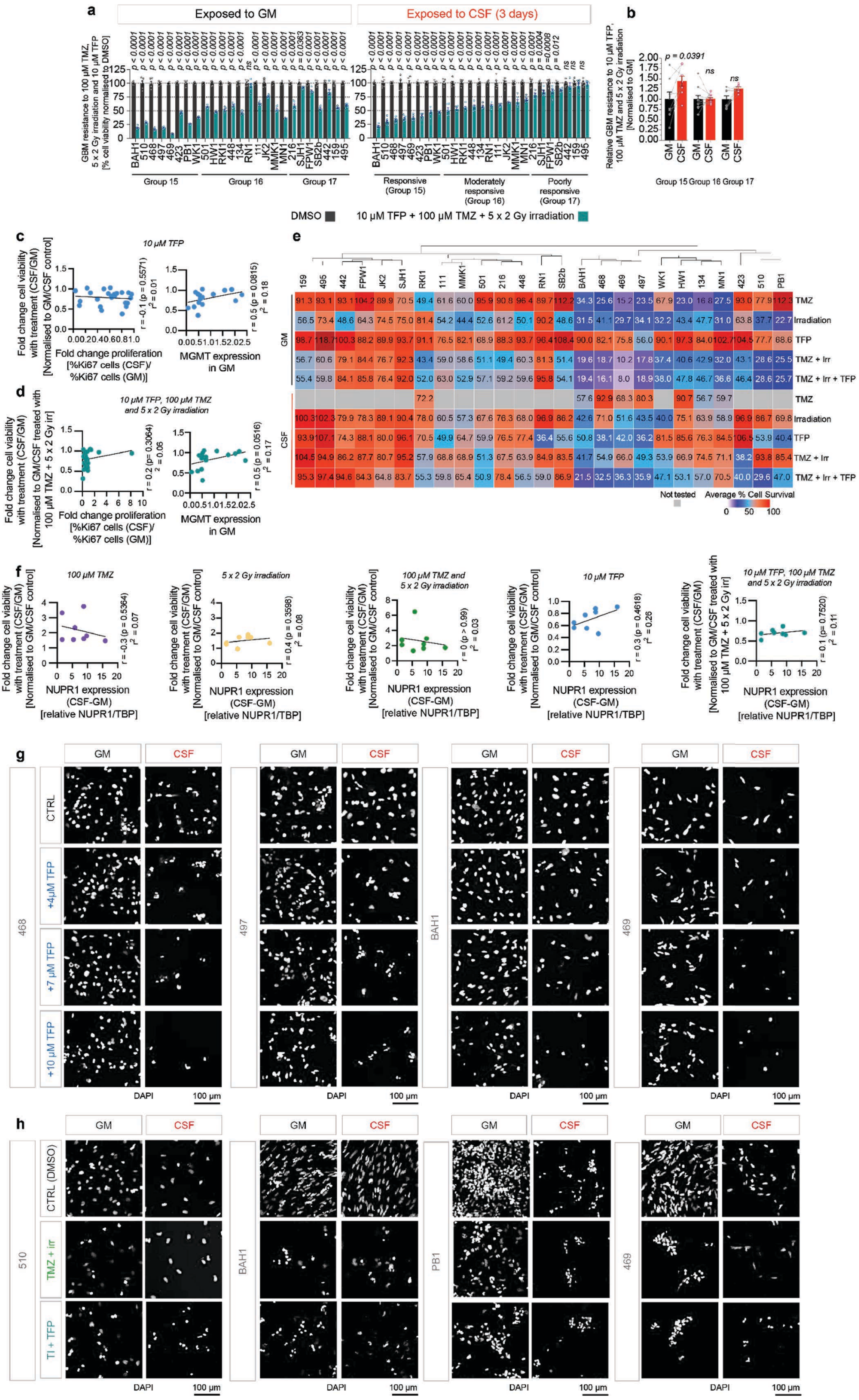

a
Exposed to GM
Exposed to CSF (3 days)
GBM resistance to 100 µM TMZ, 5 x 2 Gy irradiation and 10 µM TFP [% cell viability normalised to DMSO]
BAH1 510 468 497 469 423 PB1 WK1 501 HW1 RKI1 448 134 RN1 111 JK2 MMK1 MN1 216 SJH1 FPW1 SB2b 442 159 495
Group 15
Group 16
Group 17
Responsive (Group 15)
Moderately responsive (Group 16)
Poorly responsive (Group 17)
DMSO
10 µM TFP + 100 µM TMZ + 5 x 2 Gy irradiation
b
Relative GBM resistance to 10 µM TFP, 100 µM TMZ and 5 x 2 Gy irradiation [Normalised to GM]
p = 0.0391
ns
ns
GM CSF GM CSF GM CSF
Group 15 Group 16 Group 17
c
10 µM TFP
Fold change cell viability with treatment (CSF/GM) [Normalised to GM/CSF control]
r = -0.1 (p = 0.5571) r² = 0.01
r = 0.5 (p = 0.0815) r² = 0.18
Fold change proliferation [%Ki67 cells (CSF)/ %Ki67 cells (GM)]
MGMT expression in GM
d
10 µM TFP, 100 µM TMZ and 5 x 2 Gy irradiation
Fold change cell viability with treatment (CSF/GM) [Normalised to GM/CSF treated with 100 µM TMZ + 5 x 2 Gy irr]
r = 0.2 (p= 0.3064) r² = 0.06
r = 0.5 (p = 0.0516) r² = 0.17
Fold change proliferation [%Ki67 cells (CSF)/ %Ki67 cells (GM)]
MGMT expression in GM
e
159 495 442 FPW1 JK2 SJH1 RKI1 111 MMK1 501 216 448 RN1 SB2b BAH1 468 469 497 WK1 HW1 134 MN1 423 510 PB1
GM
TMZ: 91.3 93.1 93.1 104.2 89.9 70.5 49.4 61.6 60.0 95.9 90.8 96.4 89.7 112.2 34.3 25.6 15.2 23.5 67.9 23.0 16.8 27.5 93.0 77.9 112.3
Irradiation: 56.5 73.4 48.6 64.3 74.5 75.0 81.4 54.2 44.4 52.6 61.2 50.1 90.2 48.6 31.5 41.1 29.7 34.1 32.2 43.4 47.7 31.0 63.8 37.7 22.7
TFP: 98.7 118.7 100.3 88.2 89.9 93.7 91.1 76.5 82.1 68.9 88.3 93.7 96.4 108.4 90.0 82.1 75.8 56.0 90.1 97.3 84.0 102.7 104.5 77.7 68.6
TMZ + Irr: 56.7 60.6 79.1 84.4 76.7 92.3 43.4 59.0 58.6 51.1 49.4 60.3 81.3 51.4 19.6 18.7 10.2 17.8 37.4 40.6 42.9 32.0 43.1 28.6 25.5
TMZ + Irr + TFP: 55.4 59.8 84.1 85.8 76.4 92.0 52.0 63.0 52.9 57.1 59.2 59.6 95.8 54.1 19.4 16.1 8.0 18.9 38.0 47.8 46.7 36.6 46.4 28.6 25.7
CSF
TMZ: 72.2 57.6 92.9 68.3 80.3 90.7 56.7 59.7
Irradiation: 100.3 102.3 79.9 78.3 89.1 90.4 78.0 60.5 57.3 67.6 76.3 68.0 96.9 86.2 42.6 71.0 51.6 43.5 40.0 75.1 63.9 58.9 96.9 86.7 69.8
TFP: 93.9 107.1 74.3 88.1 80.0 96.1 70.5 49.9 64.7 59.9 76.5 77.4 36.4 55.6 50.8 38.1 42.0 36.2 81.5 85.6 76.3 84.5 106.5 53.9 40.4
TMZ + Irr: 104.5 94.9 86.2 87.7 80.7 95.2 57.9 68.8 68.9 51.3 67.5 63.9 84.9 83.5 41.7 54.9 66.0 49.3 53.9 66.9 74.5 71.1 38.2 93.8 85.4
TMZ + Irr + TFP: 95.3 97.4 94.6 84.3 64.8 83.7 55.3 59.8 65.4 50.9 78.4 56.5 59.0 86.9 21.5 32.5 36.3 35.9 47.1 53.1 57.0 70.5 40.0 29.6 47.0
Not tested
Average % Cell Survival
0 50 100
f
100 µM TMZ
r = -0.3 (p = 0.5364) r² = 0.07
5 x 2 Gy irradiation
r = 0.4 (p = 0.3598) r² = 0.08
100 µM TMZ and 5 x 2 Gy irradiation
r = 0 (p > 0.99) r² = 0.03
10 µM TFP
r = 0.3 (p = 0.4618) r² = 0.26
10 µM TFP, 100 µM TMZ and 5 x 2 Gy irradiation
r = 0.1 (p= 0.7520) r² = 0.11
Fold change cell viability with treatment (CSF/GM) [Normalised to GM/CSF control]
NUPR1 expression (CSF-GM) [relative NUPR1/TBP]
g
GM CSF
468 497 BAH1 469
CTRL
+4µM TFP
+7 µM TFP
+10 µM TFP
DAPI 100 µm
h
GM CSF
510 BAH1 PB1 469
CTRL (DMSO)
TMZ + irr
TI + TFP
DAPI 100 µm

Supplementary Fig. 10 | Targeting NUPR1 with the repurposed, antipsychotic trifluoperazine reduces cell survival in CSF. a, Percentage cell survival of 25 patient-derived GBM cell lines exposed to 1:2000 DMSO or 5 fractions of 2 Gy irradiation, 100 µM temozolomide (TMZ) and 10 µM trifluoperazine (TFP). DAPI-stained cells counted using Harmony™ software. Individual data points represent six replicates for each GBM cell line. Bar graphs represent the mean +/- SEM. Significance determined using two-way ANOVA. **b**, Fold change in percentage cell survival of cell lines responsive (Group 15), moderately responsive (Group 16), or unresponsive (Group 17) to chemoradiation (TMZ + irradiation) plus TFP. Cell viability in GM and CSF normalised to mean population survival in GM. Correlation graphs of **c,** Ki67 expression and **d,** MGMT mRNA expression against cell survival following treatment with 10 µM TFP +/- standard treatment. **e,** Heatmap and hierarchical clustering showing average response of GBM cell lines to all treatments in GM or CSF. Correlation graphs of **f,** NUPR1 mRNA expression against cell survival following standard treatment or treatment with 10 µM TFP +/- standard treatment. Difference in *NUPR1* expression calculated by subtracting *NUPR1* expression in GM from expression in CSF. **b, d, f,** Fold change of cell survival or Ki67 in CSF compared to GM was calculated by normalising treated samples untreated controls. Correlation calculated using Spearman's R and simple linear regression used to generate line of best fit. Representative images of cell lines exposed to GM or CSF and **g,** treated with DPBS, 4, 7 and 10 µM TFP for 24-hours or **h,** treated with 1:2000 DMSO, 5 x 2 Gy irradiation and 100 µM TMZ or 5 x 2 Gy irradiation, 100 µM TMZ and 10 µM TFP.

Supplementary Fig. 11

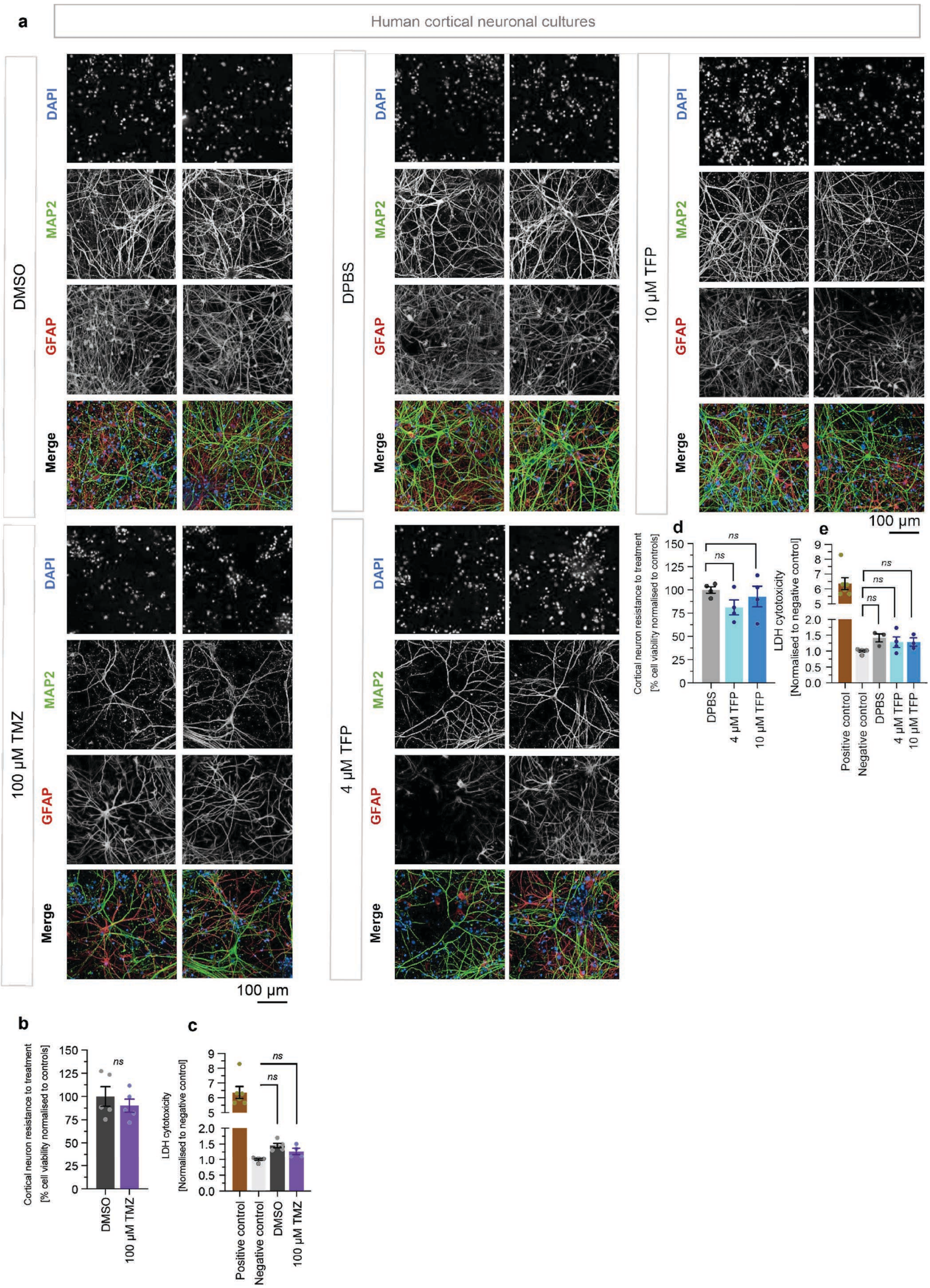

Supplementary Fig. 11 | TFP and TMZ have minimal effect on the survival of healthy human cortical neuronal cultures. a, Representative images of cortical neuronal cultures treated with DMSO, 100 μM TMZ, DPBS, 4 or 10 μM TFP. Neuronal cultures were stained for GFAP (astrocytes), MAP2 (neurons) and with DAPI (all cells). Survival of cortical neurons and astrocytes treated with **b**, 1:2000 DMSO or 100 μM TMZ or **d**, DPBS, 4 or 10 μM TFP. DAPI-stained cells counted using Harmony™ software. Individual data points represent the mean of six replicates. Error bars represent the mean +/- SEM of six replicates. Significance was determined using two-way, unpaired Mann-Whitney or Kruskal-Wallis tests. LDH cytotoxicity assays of cortical neurons and astrocytes treated with **c**, 1:2000 DMSO and 100 μM TMZ or **e**, DPBS, 4 and 10 μM TFP. LDH cytotoxicity was performed on spent media from cortical neuronal cultures. Absorbance measurements from DMSO, DPBS TMZ or TFP treated wells were normalised to the negative control composed of fresh, untreated, neuronal maturation media (NMM). Significance was determined using two-way, unpaired Kruskal-Wallis tests. ns defined as $p > 0.05$

Supplementary Fig. 12

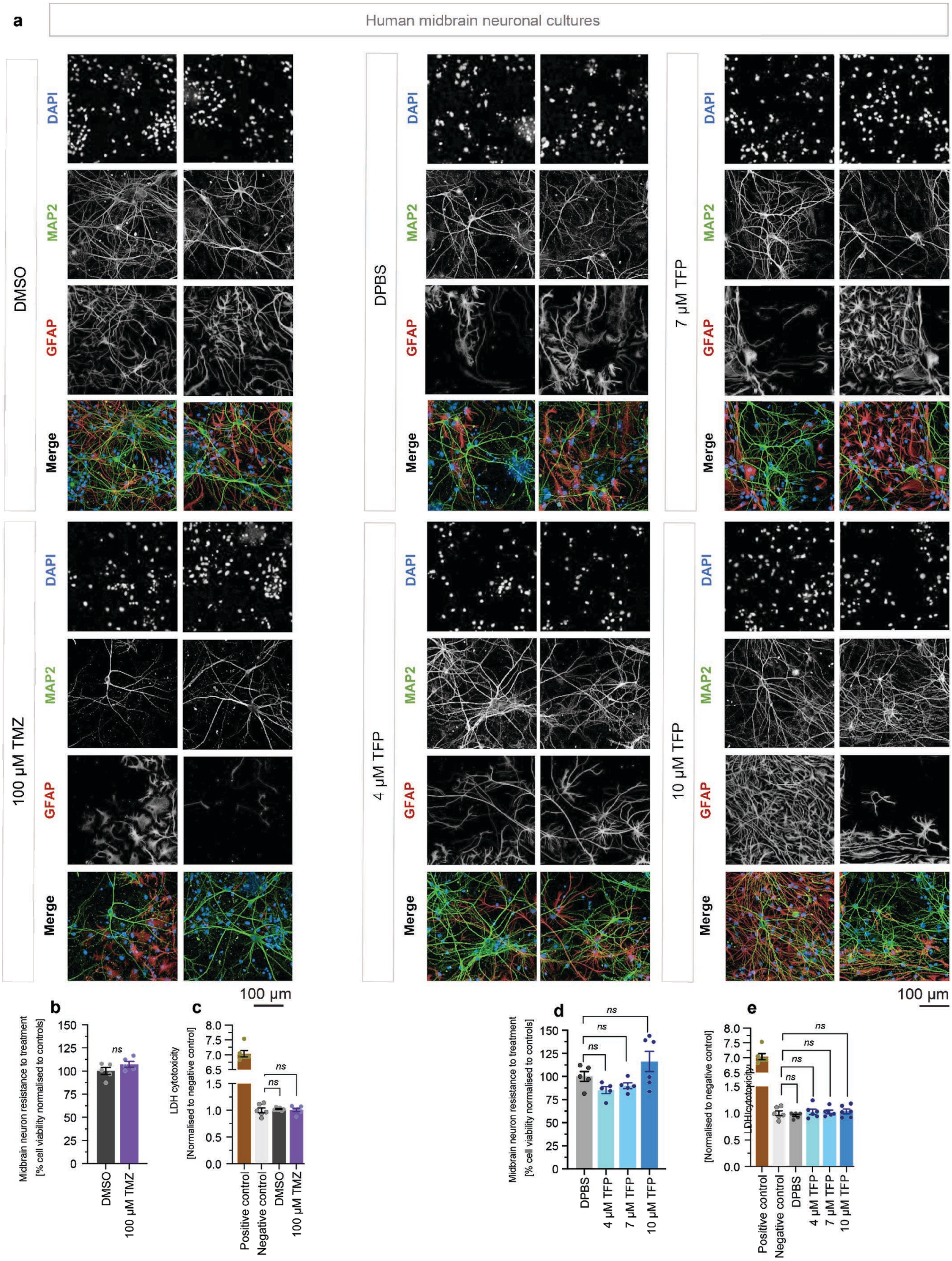

Supplementary Fig. 12 | TFP and TMZ have minimal effect on the survival of healthy human midbrain neuronal cultures. a, Representative images of midbrain neuronal cultures treated with DMSO, 100 μM TMZ, DPBS, 4, 7 or 10 μM TFP. Neuronal cultures were stained for GFAP (astrocytes), MAP2 (neurons) and with DAPI (all cells). Survival of midbrain neurons and astrocytes treated with **b**, 1:2000 DMSO or 100 μM TMZ or **d**, DPBS, 4, 7 or 10 μM TFP.DAPI-stained cells counted using Harmony™ software. Individual data points represent the mean of six replicates. Error bars represent the mean +/- SEM of six replicates. Significance was determined using two-way, unpaired Mann-Whitney or Kruskal-Wallis tests. LDH cytotoxicity assays of cortical neurons and astrocytes treated with **c**, 1:2000 DMSO and 100 μM TMZ or **e**, DPBS, 4, 7 and 10 μM TFP. LDH cytotoxicity was performed on spent media from midbrain neuronal cultures. Absorbance measurements from DMSO, DPBS TMZ or TFP treated wells were normalised to the negative control composed of fresh, untreated, neuronal maturation media (NMM). Significance was determined using two-way, unpaired Kruskal-Wallis tests. ns defined as $p > 0.05$

Supplementary Fig. 13

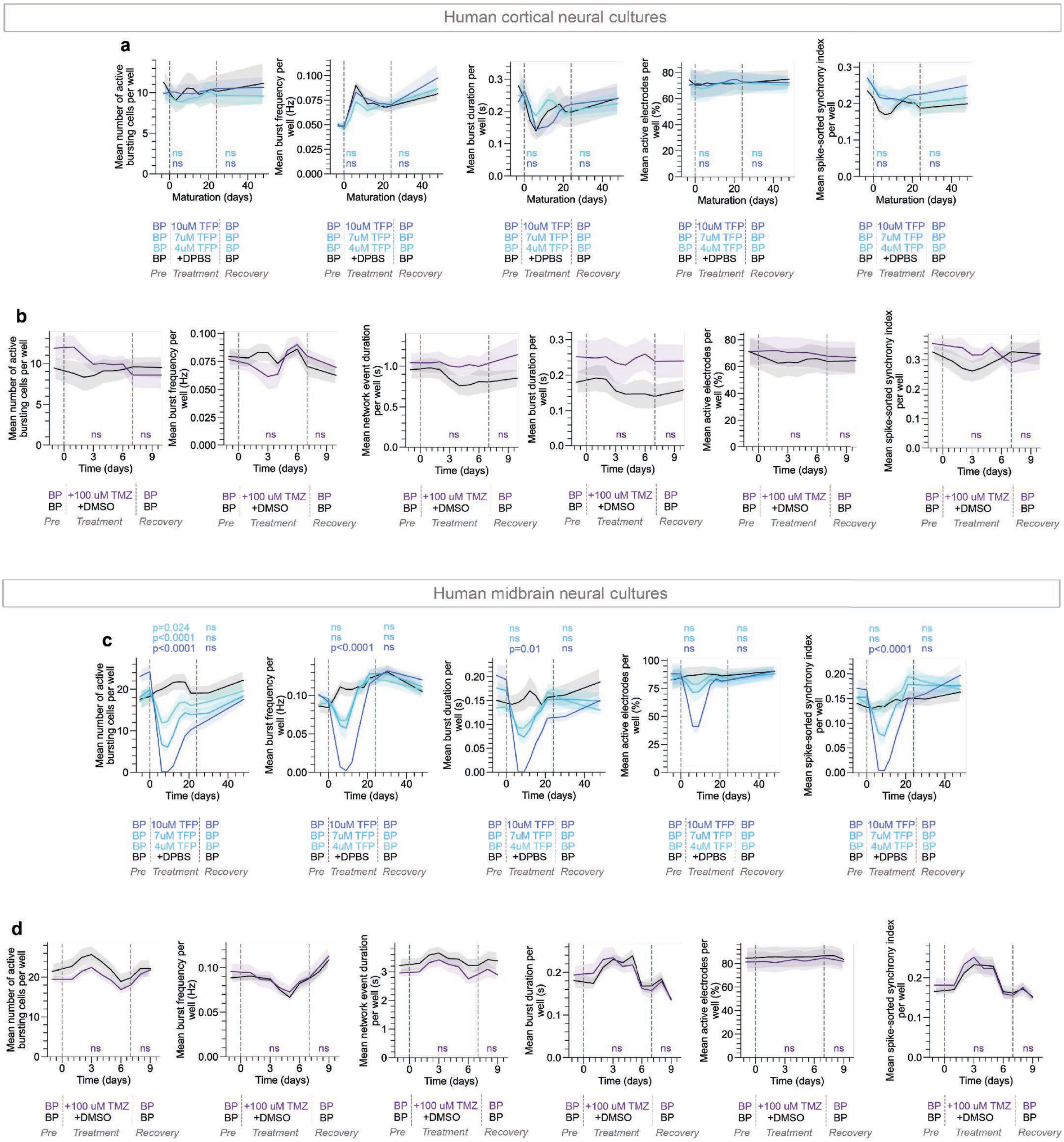

Supplementary Fig. 13 | TFP and TMZ have minimal effect on the functionality of healthy human neuronal cultures. a-d, Time series of single-cell spike-sorted neuronal electrophysiology data measured by MEA from 9 recordings over 10 days. Solid lines represent the mean of data from single neurons averaged per well smoothed with a two-point moving average algorithm, the shaded area represents +/- SEM. Dotted vertical lines indicate addition and removal of treatment. 6-9 wells analysed per condition. Area under the curve of the treatment and recovery sections was calculated in GraphPad Prism and significance was determined by unpaired t-test. **a**,**b**, Cortical neuronal cultures treated with TFP and TMZ respectively. **c**,**d**, Midbrain neuronal cultures treated with TFP and TMZ respectively. Cortical neuronal cultures for **a** had been matured in culture for 108 days at the time of treatment, cortical neuronal cultures for **b** had been matured for 100 days. Midbrain neuronal cultures for **c,** had been matured in culture for 87 days at the time of treatment, cortical neuronal cultures for **d,** had been matured for 100 days. ns defined as p > 0.05.

Supplementary Fig. 14

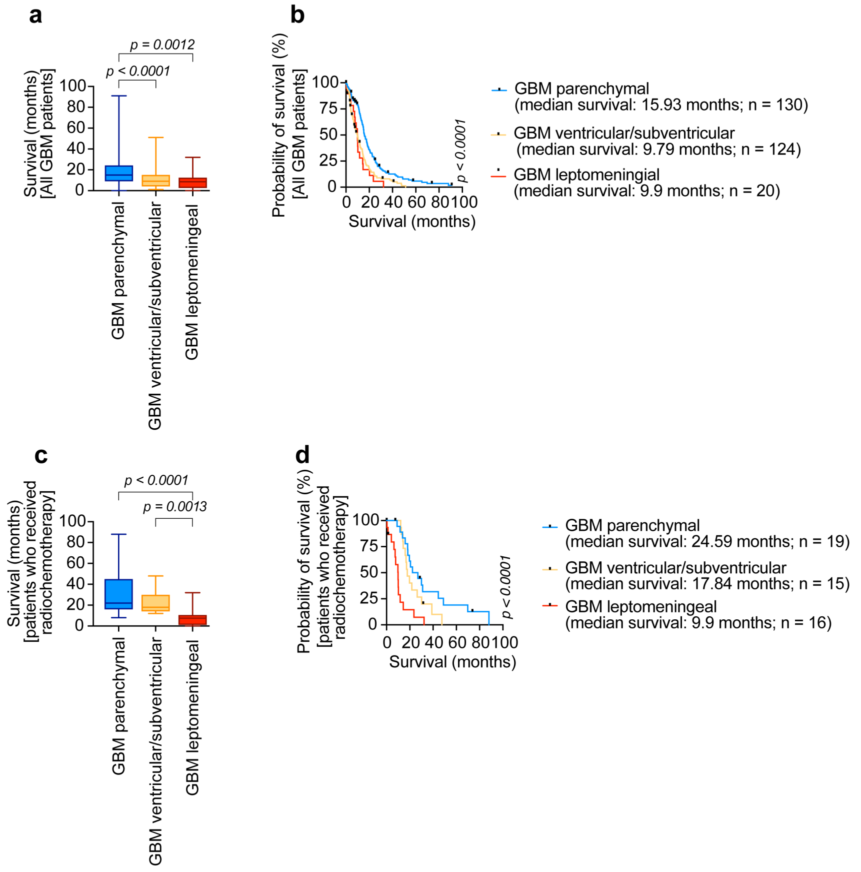

Supplementary Fig. 14 | Tumour proximity to CSF-filled ventricles or subarachnoid space correlates with lower GBM survival. Survival of GBM patients with parenchymal, ventricular and subventricular, and leptomeningeal tumours who were **a,b,** treated with radiochemotherapy or received no treatment and **c,d,** treated with standard 60 Gy irradiation and 75 mg/m2 temozolomide (with or without additional chemotherapies). Survival data were obtained from datasets published by Mistry et al, 2019 (Ref: 23) for parenchymal and ventricular/subventricular tumours and Dardis et al, 2014 (Ref: 49) for leptomeningeal disease. **a,c,** Significance was determined using non-parametric, Kruskal-Wallis test. **b**,**d,** Analysis of survival graphs was done using Kaplan-Meier and curve comparisons were conducted using logrank tests. ns defined as $p > 0.05$ and not included in graphs.

Supplementary Fig. 15

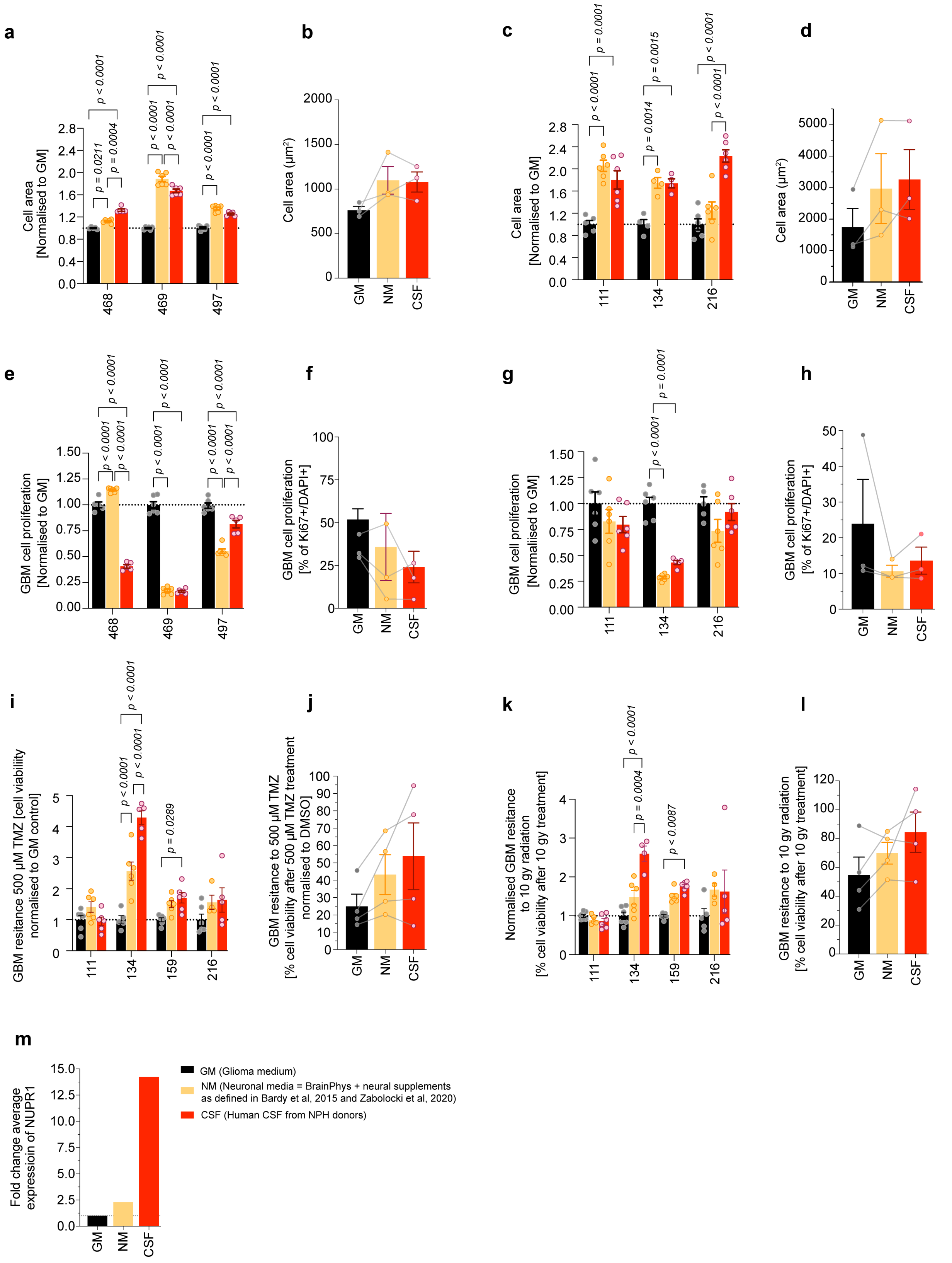

Supplementary Fig. 15 | GBM cellular phenotypes in glioma medium vs synthetic neuronal medium and human CSF. a-d, Morphology **e-h,** Proliferation or **i-l,** Cytotoxicity analysis of patient-derived GBM cells cultured in standard glioma medium (GM), a synthetic CSF (BrainPhys neuronal media; NM) or human CSF. **a,c,** Fold change of cell area normalised to GM controls. **b,d,** Paired bar-graphs show change in cell area determined using Harmony™ software analysis of CellTracker™ Deep Red or Phallodin. Cells were cultured in each medium for 4-days. **e,g,** Fold change of the percentage of proliferating cells normalised to GM controls. **f,h,** Paired bar-graphs show percentage of proliferating cells determined using Harmony™ software or ImageJ analysis of Ki67+ and DAPI+ cells. GBM cells cultured in each medium for **e,f** 4-days or **g,h** until GM condition reached confluency. **i-l,** Preliminary cytotoxicity analysis of GBM cells exposed to either **i-j,** 500 µM temozolomide (TMZ) treatment or 1:500 DMSO **k-l,** 10 gy irradiation treatment. **i,** Fold change of cell survival normalised to GM controls. **j,** Paired bar-graphs indicate percentage of cell survival normalised to DMSO controls. **k,** Fold change of cell survival normalised to GM controls. **l,** Paired bar-graphs indicate percentage of cell survival normalised to untreated controls. DAPI stained cells were analysed using ImageJ or Harmony software. **a,c,e,g,i,k** For grouped bar graphs, individual data points represent the mean +/- SEM of six replicates. Significance was determined using two-way ANOVA. **b,d,f,h,j,l** For paired bar-graphs, individual dot points represent the mean of six replicates for each cell line. Bar graphs represent mean +/- SEM of GBM cell lines. **m,** Fold change of average NUPR1 expression in SANTB00134 GBM cells cultured in GM, NM or healthy, human CSF calculated by normalising average NUPR1 expression in each condition to GM. ns defined as $p > 0.05$ and not included in graphs.

Cell line	Patient age (years)/gender	Tumour location	Tumour identity	Patient survival (days) [alive]	*IDH status*	*TERT promoter*	*CDKN2A status*	Other genetic
111	49 Female	L parietal	Primary GBM	54	WT	228C>T	Hom del	
134	58 Female	L frontal temporal	Primary GBM	[1646]	WT	228C>T	Hom del	
159	55 Male	L frontal	Primary GBM	1094	WT	WT		
216	69 Male	L frontal	Primary GBM	359	WT	WT		
423	74 Male	R parieto-occipital	Primary GBM	[169]	WT	WT		
442	49 Male	L frontal	Primary GBM	99	WT	250C>T	Hom del	
448	65 Male	L temporal	Primary GBM	130	WT	250C>T	Hom del	EgfrvIII
468	53 Female	R parietal	Primary GBM	557	WT	WT		
469	68 Male	R parieto-occipital temporal	Primary GBM	[351]	WT	228C>T		
495	67 Female	R frontal	Primary GBM	[515]	WT	WT		EgfrvIII
497	59 Female	R temporal	Recurrent GBM	[121]	WT	228C>T	Hom del	
501	63 Female	R occipital temporal	Primary GBM	477	WT	WT	Hom del	
510	73 Female	L parieto-occipital	Primary GBM	[232]	WT	228C>T		
BAH1	75 Female	R frontal	Primary GBM	94	WT	228C>T	Hom del	EgfrvIII
FPW1	68 Male	R temporal	Primary GBM	242	WT	228C>T	Hom del	
HW1	54 Female	R frontal parietal	Primary GBM	89	WT	228C>T	Hom del	
JK2	75 Male	R frontal	Primary GBM	178	WT	228C>T	Hom del	TP53 mut
MMK1	80 Female	R temporal	Primary GBM	334	WT	228C>T	Het del	
MN1	84 Female	L frontal	Primary GBM	36	WT	250C>T	Hom del	
PB1	57 Male	L frontal	Primary GBM	39	WT	250C>T	Hom del	
RKI1	57 Female	L temporal	Primary GBM	>7 years	WT	228C>T	WT	
RN1	56 Female	L temporal	Primary GBM	243	WT	228C>T	Hom del	
SB2b	48 Male	R parietal	Recurrent GBM	420	WT	250C>T	Hom del	
SJH1	72 Male	L temporal	Primary GBM	45	WT	228C>T	Hom del	Pten del
WK1	77 Male	R parietal occipital	Primary GBM	121	WT	228C>T	Hom del	Pten del

Supplementary Table 1 | Patient information for glioblastoma cell lines. This table contains patient information (age, gender, survival) for each of the 25 tumour biopsies from which the cell lines were generated. Tumour profile (location, identity and genetic aberrations) for each cell line is also depicted in this table.

CSF Batch	Patients included	Amount included	Percentage of total	Experiments	Figures	Appearance	Patient age	Patient gender	Protein concn (g/L)	Glucose concn (mM)	Osmolality (mOsm)	Reason for collection	Date collected	Bacterial growth
Batch 1														
	SACSF001	21	27.6	scRNA-seq 134	Fig. 3d-i, 4, 5, 6a	clear	NR	Female	0.13	3.5	256	LP to exclude IIH	20190619	no
	SACSF002	25	32.9			clear	88	Male	1.34	4.2	294	Suspected NPH	20190626	no
	SACSF003	12	15.8			clear	54	Female	1.01	2.8	282	Tension type headache	20190702	no
	SACSF004	18	23.7			clear	71	Female	0.37	4.5	293	NPH testing	20190806	no
Batch 2														
	Batch 1	10	16.7	scRNA-seq 111	Fig. 3d-i, 4, 5, 6a	clear								
	SACSF006	30	50			clear	26	Female	0.45	3	NM	IIH	20190828	no
	SACSF007	20	33.3			clear	66	Female	0.38	4.1	NM	NPH	20190909	no
Batch 4														
	SACSF008	5	7.7	scRNA seq 159	Fig. 3d-i, 4, 5, 6a	clear	68	Male	0.24	4.6	NM	NR	20191030	no
	SACSF009	25	38.5			clear	49	Female	0.69	4.1	NM	IIH	20191121	no
	SACSF010	15	23.1			clear	31	Male	0.24	3.6	NM	Aneurysm	20191127	no
	SACSF011	20	30.8			clear	57	Male	0.57	3.7	NM	NPH	20191127	no
Batch 7														
	SACSF027	17	75.6	TMZ dose-response 10 cell lines (Batch 6&7 combined)	Fig. 2d	blood-stained	68	Female	0.54	3.6	NM	Investigation for NPH	20210317	no
	SACSF028	5.5	24.4			clear	84	Male	0.47	3.5	NM	Investigation for NPH	20210318	no
Batch 8														
	Human CSF Batch 7	30	23.1	qPCR NUPR1 TME vs CSF	Fig. 6b,c									
	SACSF009.2	15	11.5			clear	49	Female	0.75	3.7	NM	IIH	20200702	no
	SACSF016	100	76.9			clear	34	Female	0.07	3.6	NM	IIH	20200606	no
	SACSF021	15	11.5			clear	78	Male	0.55	3.7	NM	NPH	20201008	no
Batch 9														
	Human CSF Batch 8	54.5	18.2											
	SACSF006.2	10	3.3	25 GBM cell line morphology & proliferation	Fig. 1, 3a-c	clear	28	Female	0.47	2.9	292	IIH	20210604	no
	SACSF009.3	19	6.3	25 GBM cell line TMZ, irradiation & TFP screens	Fig. 2a-g, 6f,g	NR	49	Female	NR	NR	298	IIH	NR	NR
	SACSF016	38	12.7	25 GBM cell lines TMZ + IR +/- TFP	Fig. 2f,g,i,j	clear	34	Female	0.07	3.6	290	IIH	20200606	no
	SACSF016.3	124	41.3	SB2b irradiation	Fig. 2f,g	clear	34	Female	0.13	2.7	287	IIH	20200615	no
	SACSF017	1.5	0.5	BAH1, HW1, MN1,468, 469 & 497 scRNA-seq	Fig. 3d-i, 4, 5, 6a	NR	78	Male	NR	NR	290	Communicating hydrocephalus	20200611	no
	SACSF022	20	6.7			clear	79	Male	0.4	4.2	287	NR	20201012	no
	SACSF029	6	2			clear	63	Female	0.21	3.2	266	Post-op CSF leak (pituitary adenoma)	20210428	no
	SACSF030	17	5.7			clear	76	Female	0.18	3.2	292	NPH	20210428	no
	SACSF033	10	3.3			clear	20	Female	0.51	3.1	289	IIH	20210628	no
Batch 10														
	SACSF041	20	52.6	BAH1, 468,469, 497 TFP dose-response TME vs CSF	Fig. 6i	clear	27	Male	0.38	3.6	NM	Congenital hydrocephalus	20211112	no
	SACSF043	18	47.4			clear	54	Female	0.54	5.6	304	IIH	20211203	no

NM ot measurable
NR not reported

Supplementary Table 2 | Information of cerebrospinal fluid samples. This table contains patient information (age, gender, health status, volume received) alongside the cerebrospinal fluid profile (CSF; protein concentration, osmolality and glucose) for CSF samples used in this study. A unique CSF identification (SACSF number) was given to each patient upon collection. A single CSF batch contains multiple patient CSF samples combined for experiments.

Supplementary Table 3. Key materials and resources used.

Reagent or resource	Source	Identifier
**Antibodies/imaging markers**		
Anti-Ki67 (rabbit polyclonal)	Abcam	Cat # ab15580
Anti-MAP2 (chicken polyclonal)	Abcam	Cat # ab5392
Anti-GFAP (mouse monoclonal)	Abcam	Cat # ab4648
Secondary antibody (donkey anti-chicken Alexa Fluor™ 488)	Jackson ImmunoResearch	Cat # 703-545-155
Secondary antibody (donkey anti-rabbit Alexa Fluor™ 594)	Abcam	Cat # ab150068
Secondary antibody (donkey anti-mouse Alexa Fluor™ 647)	Abcam	Cat # ab150111
Phalloidin-iFluor™ 488	Abcam	Cat # ab176753
CellTracker™ Deep Red	Abcam	Cat # C34565
DAPI	Sigma-Aldrich	Cat # D9542-1MG
**Media reagents**		
DMEM/F12 GlutaMax	ThermoFisher	Cat # 10565018
NeuroCult SM1 neuronal supplement with vitamin A	STEMCELL Technologies	Cat # 05711
NeuroCult SM1 neuronal supplement without vitamin A	STEMCELL Technologies	Cat # 05731
N2 supplement-A	STEMCELL Technologies	Cat # 07152
Human recombinant EGF	STEMCELL Technologies	Cat # 78136
Human recombinant FGFbasic	Thermo Fisher scientific	Cat # 78134
Human recombinant FGF8b	PeproTech	Cat # 100-25
Ascorbic acid	Sigma	Cat # A4403
Sonic hedgehog	PeproTech	Cat # 100-45
BDNF	STEMCELL Technologies	Cat # 78133.1
GDNF	STEMCELL Technologies	Cat # 78139.1
Dibutyryl cyclic-AMP	Sigma	Cat # D0627
Reduced growth factor Matrigel	Corning	Cat. # CLS356230
Poly-L-ornithine	Sigma	Cat #P3655
Laminin	Life Technologies	Cat #23017015
Accutase™	STEMCELL Technologies	Cat #07920
Human cerebrospinal fluid	South Australia Cerebrospinal Fluid Biobank	
**Drugs and Chemicals**		
Temozolomide	Sigma-Aldrich	Cat # T2577
Trifluoperazine	Sigma-Aldrich	Cat # T6062-5G
Paraformaldehyde	Sigma-Aldrich	Cat # P6148-500G
**Single cell RNA seq reagents**		
3' CellPlex Kit Set A	10X Genomics	Cat #1000261
**Experimental models: cell lines**		
SANTB00111	Ebert	
SANTB00134	Ebert	
SANTB00159	Ebert	
SANTB00216	Ebert	
SANTB00423	Bardy lab	
SANTB00442	Bardy lab	
SANTB00448	Bardy lab	
SANTB00468	Bardy lab	
SANTB00469	Bardy lab	

SANTB00495	Bardy lab	
SANTB00497	Bardy lab	
SANTB00501	Bardy lab	
SANTB00510	Bardy lab	
BAH1	Q-Cell	QIMR-B001
FPW1	Q-Cell	QIMR-B002
HW1	Q-Cell	QIMR-B003
JK2	Q-Cell	QIMR-B004
MMK1	Q-Cell	QIMR-B005
MN1	Q-Cell	QIMR-B006
PB1	Q-Cell	QIMR-B007
RKI1	Q-Cell	QIMR-B008
RN1	Q-Cell	QIMR-B009
SB2b	Q-Cell	QIMR-B010
SJH1	Q-Cell	QIMR-B011
WK1	Q-Cell	QIMR-B012
WA09	WiCell	
**Commercial kits**		
Tumor Dissociation Kit (human)	Miltenyi Biotec	Cat #130-095-929
**Commercial assays**		
Cytotox96 Non-Radioactive Cytotoxicity Assay Kit	Promega	Cat # G1780
